# The substrate envelope strategy: Structural basis for resistance mitigation in antiviral drug design

**DOI:** 10.1016/j.pscia.2026.100147

**Published:** 2026-09-04

**Authors:** Qiaojie Xu, Jiwei Zhang, Yanxu Hou, Zhou Tan, Heng Gao, Edeildo F. Silva-Júnior, Shenghua Gao, Peng Zhan, Xinyong Liu

**Affiliations:** a Department of Medicinal Chemistry, State Key Laboratory of Discovery and Utilization of Functional Components in Traditional Chinese Medicine, Shandong Key Laboratory of Druggability Optimization and Evaluation for Lead Compounds, Shandong Basic Science Special Academic Zone (Pharmacy), School of Pharmaceutical Sciences, Shandong University, 44 West Culture Road, Jinan, Shandong, 250012, PR China; b Research Group of Biological and Molecular Chemistry, Institute of Chemistry and Biotechnology, Federal University of Alagoas, Lourival Melo Mota Avenue, AC. Simões campus, Alagoas, Maceió, 57072-970, Brazil; c China-Belgium Collaborative Research Center for Innovative Antiviral Drugs of Shandong Province, 44 West Culture Road, Jinan, Shandong, 250012, PR China

**Keywords:** Antiviral drugs, Drug resistance, Substrate envelope hypothesis, Structure-based drug design

## Abstract

Drug resistance remains a major hurdle in antiviral drug development. This review highlights the “substrate envelope hypothesis”, a structure-based drug design (SBDD) paradigm that constrains the spatial boundaries and binding footprint of inhibitors within the conserved active site volume defined by natural viral substrates. By restricting inhibitor interactions to evolutionarily conserved residues, this approach ensures that mutations abrogating drug binding simultaneously impair natural substrate processing. This imposes an insurmountable fitness cost on the virus, thereby mitigating resistance. Through the analysis of viral enzyme-substrate co-crystal structures, we systematically review the application of this framework in human immunodeficiency.virus (HIV) protease, hepatitis C virus (HCV) NS3/4A protease, influenza virus (IFV) neuraminidase (NA), and severe acute respiratory Severe Acute Respiratory Syndrome Coronavirus 2 (SARS-CoV-2) main protease, illustrating how substrate envelope-guided design achieves high potency while circumventing resistance. Additionally, we extend this strategy from traditional protease targets to non-protease enzymes. By integrating molecular dynamics and structural biology, this framework offers a rational approach for developing next-generation antivirals.

**Table:** List of Abbreviations

Abbreviation	Full Term
ATV	Atazanavir
APV	Amprenavir
CoVs	Coronaviruses
DAAs	Direct-acting Antivirals
DRV	Darunavir
DTG	Dolutegravir
EVG	Elvitegravir
FDA	Food and Drug Administration
HBV	Hepatitis B Virus
HCV	Hepatitis C Virus
HIV	Human Immunodeficiency Virus
IDV	Indinavir
IFV	Influenza Virus
IN	Integrase
LPV	Lopinavir
M^pro^	Main Protease
MST	Microscale Thermophoresis
NA	Neuraminidase
NAIs	Neuraminidase Inhibitors
NFV	Nelfinavir
PA	Polymerase Acidic Protein
RAL	Raltegravir
PIs	Protease Inhibitors
PR	Protease
RdRp	RNA-dependent RNA polymerase
RT	Reverse Transcriptase
RTV	Ritonavir
SAR	Structure-activity Relationships
SARS-CoV-2	Severe Acute Respiratory Syndrome Coronavirus 2
SBDD	Structure-based drug design
SQV	Saquinavir
TPV	Tipranavir
vdW	van der Waals

## 1 Introduction: drug resistance to antiviral drugs

Viral infections, including COVID-19, HIV, HCV, IFV, and HBV, continue to impose a profound global health burden, underscoring the urgent need for innovative therapeutics [[1], [2], [3], [4], [5], [6]]. However, the extensive genetic heterogeneity of these viruses—ranging from distinct HCV genotypes and HIV clades to strain-specific IFV variations—presents a formidable barrier to long-term treatment success [[7], [8], [9]]. Even when an antiviral is potent against a particular wild-type virus strain, resistance can develop. Under pharmacologic selective pressure, resistant variants with amino-acid substitutions at the drug-binding site gain a replicative advantage and become dominant. In the absence of antiviral medication, host immune responses can drive the evolution of viral strains, potentially selecting for more virulent or transmissible phenotypes [[10], [11], [12]]. Consequently, viral resistance and mutational escape remain formidable obstacles that substantially erode the clinical efficacy of antiviral agents, representing a pervasive challenge in antiviral drug discovery and development.

**Fig. 1:**
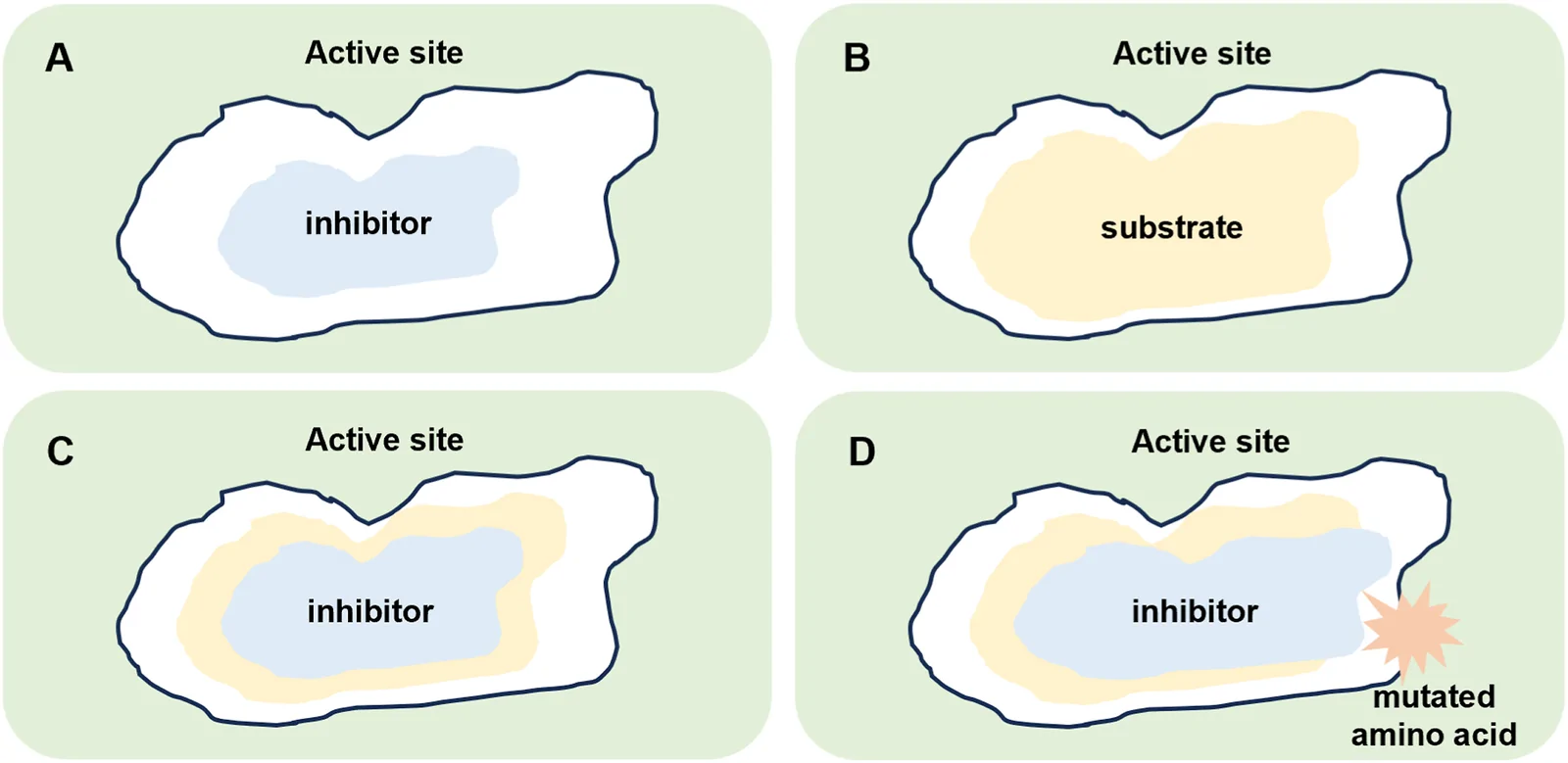
Illustration of the substrate envelope hypothesis. (**A** and **B**) Depiction of the binding occupancy patterns for both substrate and inhibitor within the enzyme's active site, highlighting their spatial relationships and interactions; (**C**) Demonstration of how an inhibitor's binding to the substrate's region within the active site can increase the target's mutational difficulty, thereby potentially reducing the likelihood of resistance development; (**D**) Visualization of a scenario where the drug target has undergone mutation in a region that is in direct contact with the inhibitor, potentially leading to changes in binding affinity and resistance emergence.

Structure-based drug design (SBDD) remains the most fundamental and most efficacious paradigm in contemporary antiviral discovery pipelines [13]. While conventional strategies—such as targeting highly conserved residues, optimizing hydrogen-bonding networks, and multi-target design—can improve the genetic barrier to resistance [[14], [15], [16], [17]], they predominantly prioritize binding enthalpy and entropy. By frequently neglecting viral evolutionary dynamics and target heterogeneity, these traditional affinity-driven approaches remain susceptible to mutational evasion [18,19]. Primary resistance mutations typically emerge at the drug-binding site, evolutionarily favoring substrate recognition over drug affinity. To address this bottleneck, Celia Schiffer's group demonstrated in 2002 that HIV-1 protease recognizes substrates via a conserved spatial conformation rather than specific amino acid sequences [20], formally coining this consensus volume the “substrate envelope” in 2006 [21]. The substrate envelope defines a conserved, asymmetric three-dimensional cavity within the active site, mapped by overlapping the binding conformations of all natural substrates processed by the enzyme (Fig. 1A and B) [22]. Because the active site residues delineating this envelope are indispensable for catalytic function, mutations at these positions severely impair substrate processing and viral viability [[23], [24], [25]]. Consequently, small-molecule inhibitors fully embedded within the boundaries of this conserved envelope (Fig. 1C) interact exclusively with evolutionarily invariant residues. Any mutation disrupting drug binding will simultaneously abolish natural substrate cleavage, imposing a lethal fitness cost on the virus. Conversely, inhibitors protruding beyond the envelope (Fig. 1D) interact with non-conserved residues irrelevant to substrate recognition; mutations here readily abrogate drug binding without compromising substrate catalysis, yielding viable resistant strains. Ultimately, constraining inhibitor occupancy within the substrate envelope establishes a robust genetic barrier against viral mutational escape.

Integrating SBDD with substrate-envelope guided drug design, allows the rational sculpting of inhibitors that maximize complementarity to the conserved catalytic cavity while minimizing protrusion beyond the envelope. This article provides a comprehensive overview of the state-of-the-art development and application of the substrate envelope hypothesis in the design of inhibitors targeting HIV, HCV, IFV and SARS-CoV-2. It offers new insights for the contemporary design of antiviral drugs.

## 2 Human immunodeficiency virus (HIV)

**Fig. 2:**
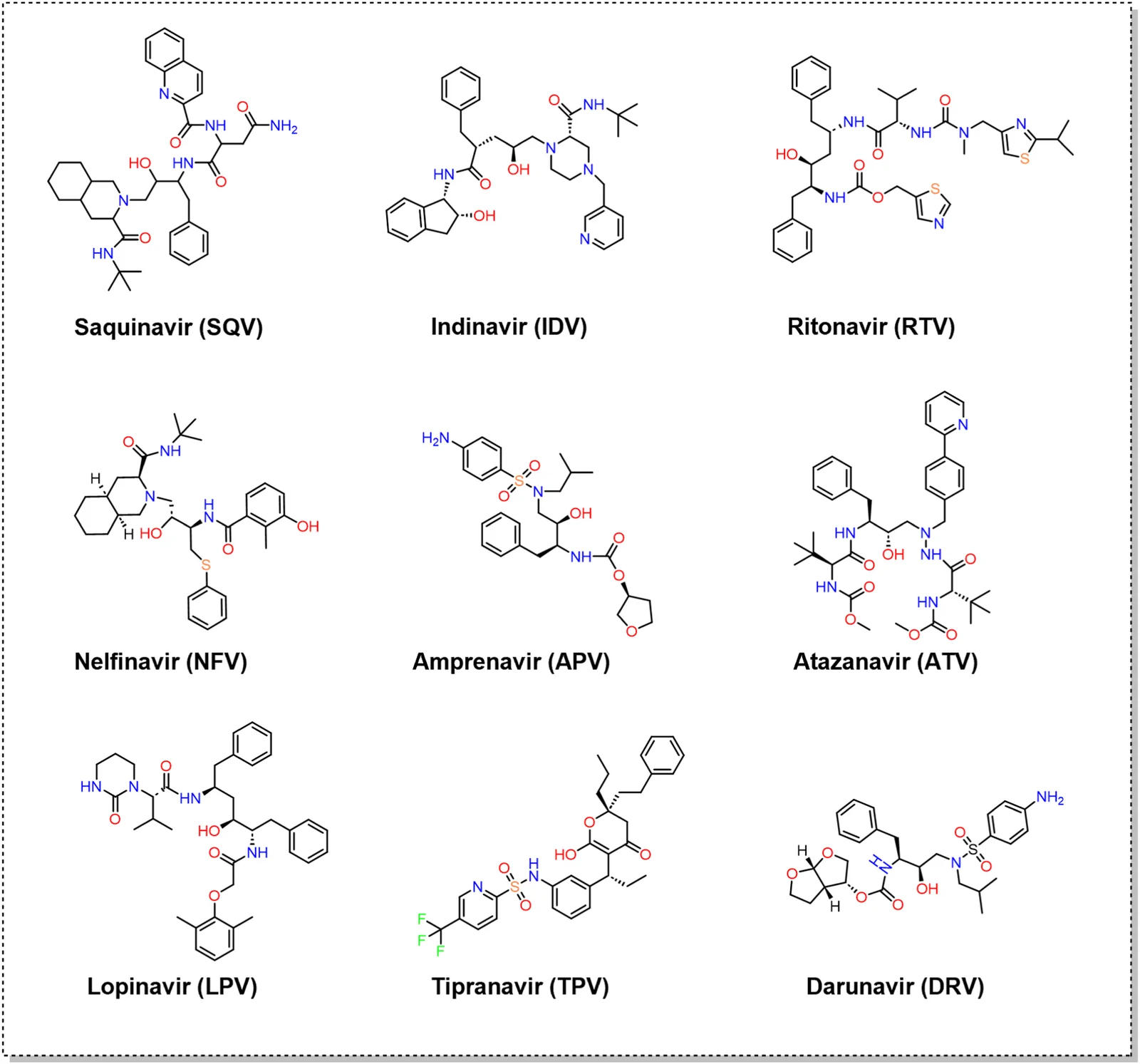
Chemical structures of FDA-approved HIV-1 PR inhibitors.

Currently, there are approximately 39 million individuals worldwide living with HIV-1, and the United States experiences around 40,000 new infections annually [26]. Direct-acting antivirals (DAAs) target essential viral enzymes—reverse transcriptase (RT), integrase (IN), and protease (PR)—that orchestrate the HIV-1 life cycle. HIV protease (PR), plays an essential role in the maturation process of HIV-1. It can cleave the precursor polyproteins Gag and Gag-pol, thereby releasing peptides within various proteins and enzymes, which completes the viral life cycle. Its inhibition arrests viral maturation and confers potent antiviral activity. HIV PR inhibitors exemplify the inaugural success of structure-based drug design (SBDD). Currently, there are nine FDA-approved inhibitors for HIV-1 PR (also, known as retropepsin), namely indinavir (IDV), nelfinavir (NFV), amprenavir (APV), saquinavir (SQV), ritonavir (RTV), lopinavir (LPV), atazanavir (ATV), tipranavir (TPV), and darunavir (DRV) (Fig. 2) [[27], [28], [29], [30], [31], [32], [33], [34]]. All these drugs are competitive inhibitors. Despite theirshared mechanism of action, they display distinct yetoverlapping resistance-associated substitution patterns, ultimately yielding multidrug-resistant PR variants.

By mapping atomic-resolution contacts between HIV-1 PR and its diverse non-homologous substrates, Schiffer's research group delineated an overlapping substrate-binding locus, the substrate envelope, central to substrate recognition [35]. This envelope presents an asymmetric topography: a toroidal architecture on the unprimed flank and an extended configuration on the primed flank. This asymmetric region plays a pivotal role in defining the sequence-specific recognition motif through which HIV-1 PR discriminates viable scissile substrates. In fact, primary active site drug-resistant mutations in HIV PR are localized precisely at the interface where inhibitors extend beyond the substrate envelope. To interrogate this paradigm, the team developed various novel inhibitors to evaluate the substrate envelope hypothesis. These inhibitors were designed based on the (*R*)-(hydroxyethylene) sulfonamide isostere, which bears resemblance to APV and DRV. The selection of this scaffold was driven by its predominant fit within the substrate envelope, synthetic accessibility, and having three sites for substituent modification. Systematic substitution at these three positions generated analogs evaluated against a panel of multidrug-resistant HIV-1 PR variants, thereby testing the substrate-envelope hypothesis.

**Fig. 3:**
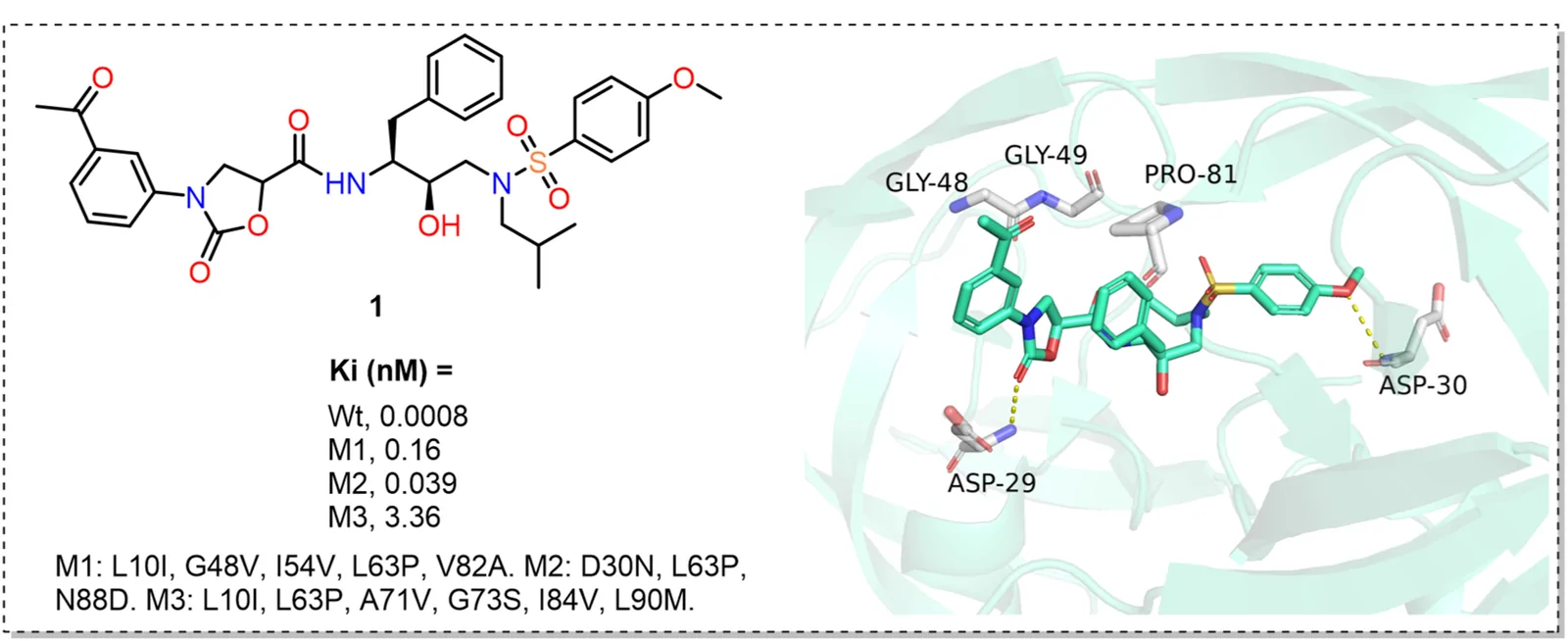
Chemical structure of compound **1** (green) in the active site of HIV-1 PR (PDB code: 2I0D). (For interpretation of the references to colour in this figure legend, the reader is referred to the Web version of this article.)

In 2006, they designed a series of inhibitors with structural variations at the P2 phenyloxazolidinone and P2′ phenylsulfonamide moiety [36]. Among these compounds, those bearing 3-acetyl, 4-acetyl, and 3-trifluoromethyl substituents on the oxazolidinone phenyl ring demonstrated exceptional potency within their series, exhibiting *K*_*i*_ values in the low picomolar (pM) range. The most potent protease inhibitor, designated as compound **1**, initially displayed an extremely high affinity for the wild-type enzyme (*K*_*i*_ = 0.8 pM), and it retained potent binding activity against resistant variants (M1: L10I, G48V, I54V, L63P, V82A; M2: D30N, L63P, N88D; M3: L10I, L63P, A71V, G73S, I84V, L90M) with *K*_*i*_ values ranging from picomolar to low nanomolar. Crystal structure analysis of compound **1** in complex with wild type HIV-1 PR provided key insights into its molecular interactions (Fig. 3). Specifically, the carbonyl group of the oxazolidinone ring forms hydrogen bonds with the highly conserved Asp29 residue of this protease. In addition, at the P2’ end of compound **1**, the methoxyl group establishes a hydrogen-bonding interaction with the backbone nitrogen atom of Asp30 residue in the protease. Furthermore, van der Waals (vdW) contacts are observed involving between the carbonyl group of 3-acetyl moiety in compound **1** and the residues Gly48, Gly49, and Pro81. These interactions underscore the effectiveness of compound **1** in binding to HIV-1 PR, even in the presence of resistance-conferring mutations.

**Fig. 4:**
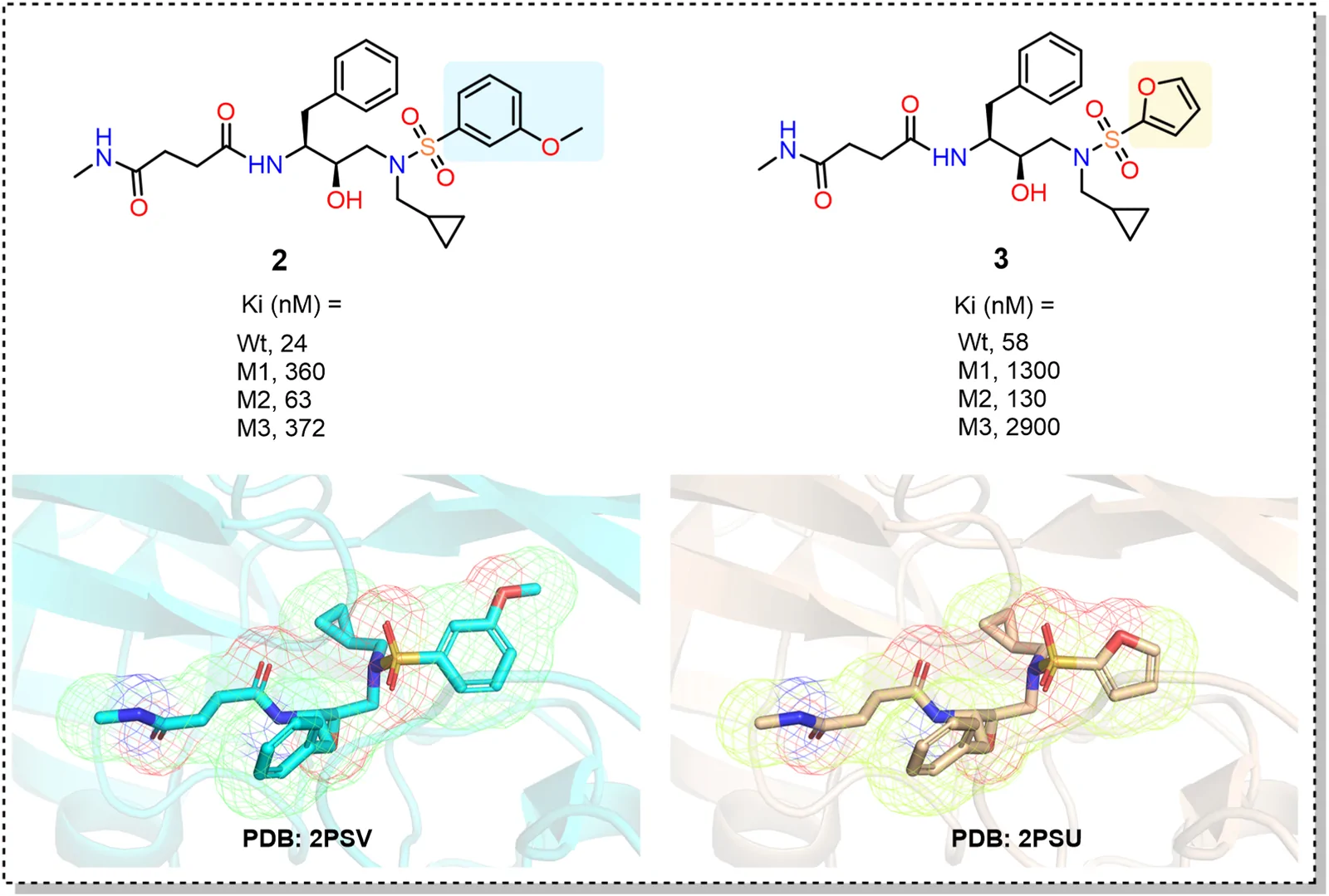
Chemical structures of compounds **2** and **3**. Binding modes of compound **2** (cyan) (PDB code: 2PSV) and **3** (golden) (PDB code: 2PSU) at the active site of HIV-1 PR. (For interpretation of the references to colour in this figure legend, the reader is referred to the Web version of this article.)

In 2007, an extensive optimization of docking studies was carried out to generate a combinatorial library of compounds with significant structural variation at three key positions, aiming to identify molecules that could both fit within the substrate envelope and exhibit high-affinity binding [37]. The candidate compounds were evaluated based on their predicted binding affinity from the docking simulations, as well as their proximity to the substrate envelope for inhibitor fitting. A genetic algorithm (GA) was then applied to identify the optimal combinations of substituents, resulting in the creation of a targeted library of a targeted compound library. Among the resulting inhibitors, compound **2** demonstrated the strongest binding affinity, with a value of 24 nM (Fig. 4). Importantly, this inhibitor showed a consistent binding profile against resistant variants compared to NFV, LPV, IDV, SQV and RTV, compound **2** displayed a more consistent binding profile across resistant variants compared to NFV, LPV, IDV, SQV, and RTV. Specifically, compound **2** showed a maximum 15-fold decrease in affinity in mutant strains, whereas compound **3** exhibited a 50-fold decrease. However, both compounds displayed relatively weaker affinities compared to APV. Correspondingly, the calculated volumes by which compound **2** and **3** protrude beyond the substrate envelope are slightly greater than that of APV, yet smaller than those of NFV, LPV, IDV, SQV, and RTV. Furthermore, co-crystal structures confirmed that these inhibitors fit within the designed substrate envelope (Fig. 4).

**Fig. 5:**
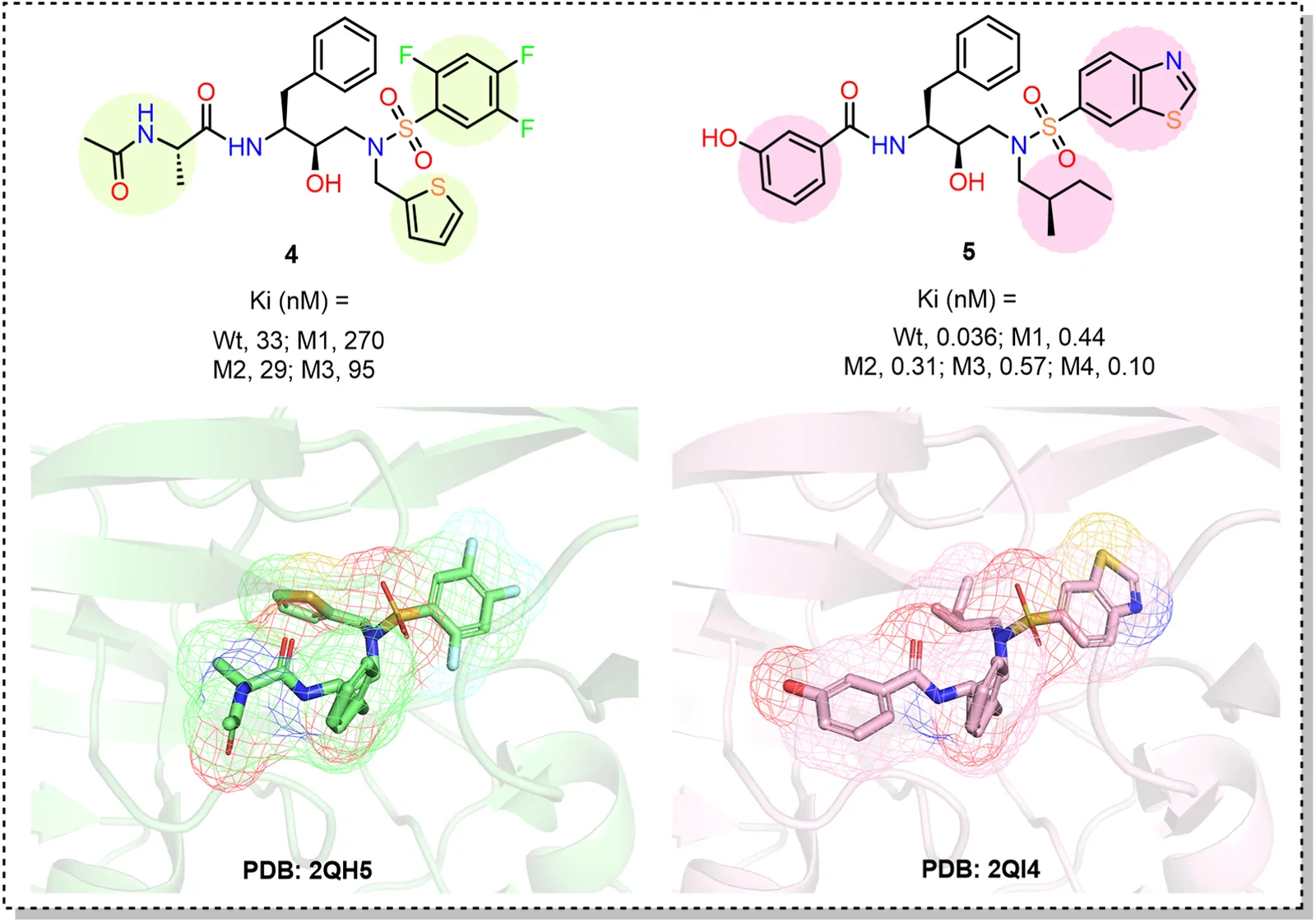
Chemical structures of compounds **4** and **5**. Binding modes of compounds **4** (green) (PDB code: 2QH5) and **5** (pink) (PDB code: 2QI4) at the active site of HIV-1 PR. (For interpretation of the references to colour in this figure legend, the reader is referred to the Web version of this article.)

In 2008, a computer-aided inverse molecular design method incorporating small-molecule design was utilized to identify molecules that fit within the substrate envelope and complement the active site of HIV PR. This approach was implemented in two phases: design and synthesis [38]. The first round yielded inhibitors with binding affinities varying from 26 μM to 30 nM (compound **4**, *K*_*i*_ = 33, 270, 29, 95 nM upon HIV-1 PR wild-type, M1, M2, M3, respectively) (Fig. 5), exhibiting a consistent binding profile. The most potent inhibitors showed no more than an 8-fold reduction in affinity across a panel of resistant variants. Subsequently, a second, re-optimized library was designed based on the high affinity inhibitor-protease complex rather than the substrate-protease complex. This strategy yielded inhibitors with significantly enhanced binding affinities, ranging from 4.2 nM to 36 pM. A subset of those inhibitors retained robust flat sub nanomolar binding affinity to a panel of resistant variants (compound **5**, *K*_*i*_ = 0.036, 0.44, 0.31, 0.57, 0.10 nM against HIV-1 PR wild-type, M1, M2, M3, M4, respectively). Moreover, co-crystal structures of the inhibitors confirmed their close alignment with the substrate envelope (Fig. 5), further supporting the potential of substrate envelope-based design as a strategy for developing HIV-1 PR inhibitors with reduced resistance potential.

In 2011, Schiffer's group carried out molecular dynamics simulations on seven distinct HIV-1 PR-substrate complexes, covering five cleavage sites in the Gag polyprotein and two in the Gag-Pro-Pol polyprotein [39]. By incorporating dynamic information into the substrate envelope framework, they demonstrated that substrate recognition relies on conformational dynamics rather than static structures, thus refining the original static substrate envelope theory. This research also uncovered an intramolecular compensatory interaction network within substrates: reduced binding at one position can be offset by strengthened contacts at other positions, keeping the overall binding energy around −45 kcal/mol. Such a mechanism accounts for the broad recognition of divergent substrate sequences by a single protease. These results support a new design strategy in which the dynamic substrate envelope serves as a strict structural constraint to minimize inhibitor protrusion and avoid interactions with resistance-associated residues. Accordingly, rational drug design should target conformational ensembles instead of individual static crystal conformations. Nevertheless, their simulations were limited to the catalytically inactive D25N PR variant. Further investigations can be expanded to clinically relevant resistant PR-substrate complexes to support the development of resistance-overcoming inhibitors.

**Fig. 6:**
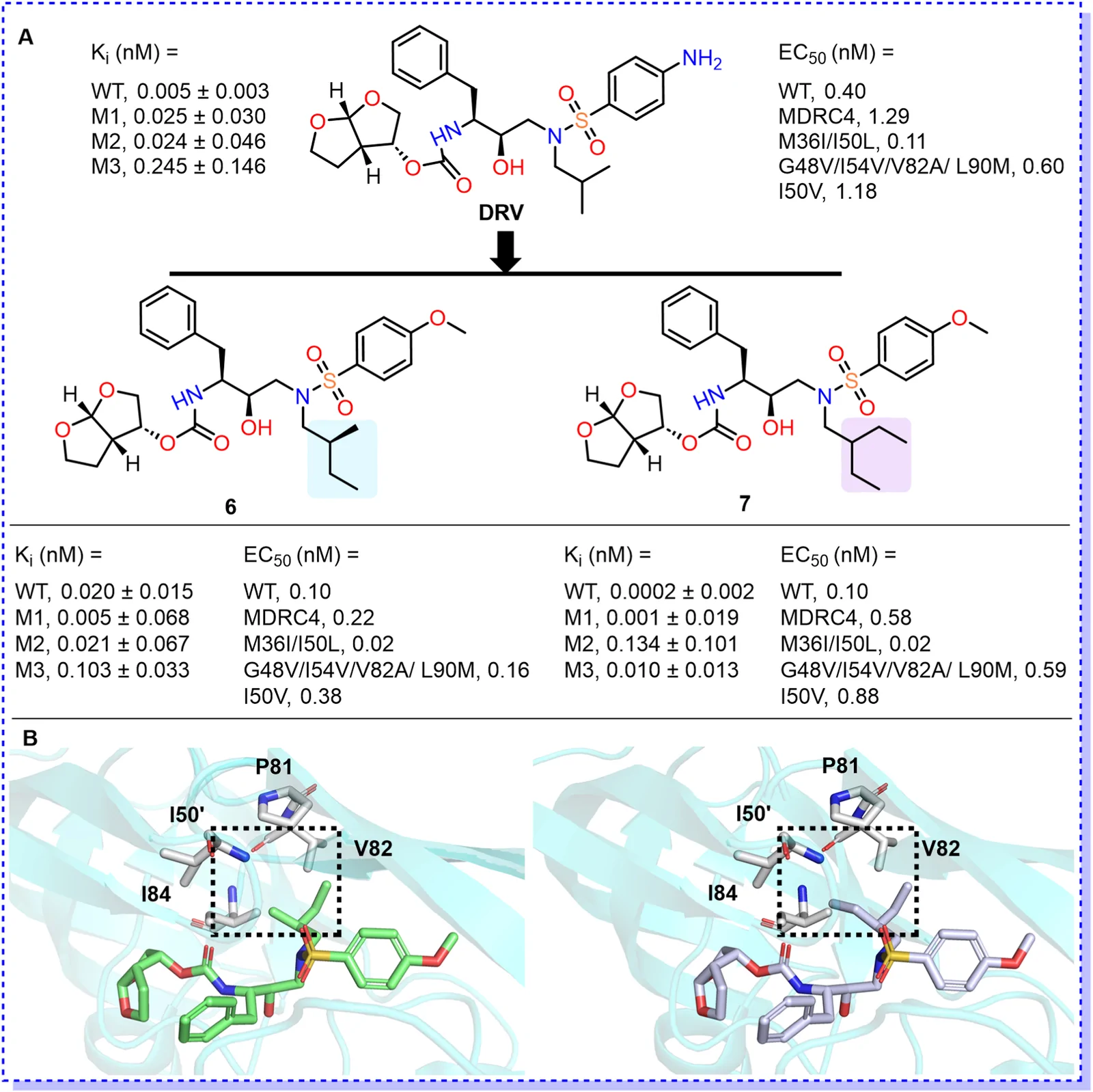
Discovery of novel DRV derivatives **6** and **7**. (A) Chemical structures and activities of compounds **6** and **7**. (B) Binding modes of **6** (limegreen) (PDB code: 3O9A) and **7** (lightblue) (PDB code: 3O9F) occupied at the active site.

Previous studies have suggested that the interactions in S1′ and S2′ subsites of PR can be enhanced by incorporating more hydrophobic P1’ (the isobutyl region in APV) and P2′ groups, thereby facilitating vdW contacts with protease variants harboring active site mutations. In 2013, a substrate envelope strategy was employed to refine protease inhibitors (PIs) based on the DRV framework. This approach involved integrating ligands at the P1′ and P2′ positions to closely mimic the natural substrate envelope, leading to the identification of promising candidates capable of potently inhibiting multidrug-resistant (MDR) HIV-1 PR variants [40]. Researchers explored two suitable substituents like isopentyl and isohexy groups that were flexible and had proper volume at P1′ position, resulting in compounds **6** and **7** respectively (Fig. 6A). These compounds were found to fit well within the substrate envelope and enhance the vdW interactions between inhibitors with proteases compensating for the loss of interaction between the *iso*-butyl of DRV with mutants I50V, V82A and I84V. All designed inhibitors demonstrated high potency in suppressing the enzyme's activity, with *K*_*i*_ values in the 0.2-30 pM range against wild-type PR, while the *K*_*i*_ value of the most effective FDA-approved DRV was 5 pM, suggesting that several new designed compounds may show greater effect against wild-type than DRV. Besides, these inhibitors also showed excellent potency against MDR, as for the M1, M2 and M3 PR of MDR, they showed even greater potency compared with DRV. The longer isopentyl, and isohexy groups were found to fit more effectively into the hydrophobic pocket S1, which contains hydrophobic residues Ile50, Pro81, Val82, and Ile84 of PR, and the groups still maintained vdW interactions with mutants I50V, V82A and I84V (Fig. 6B).

**Fig. 7:**
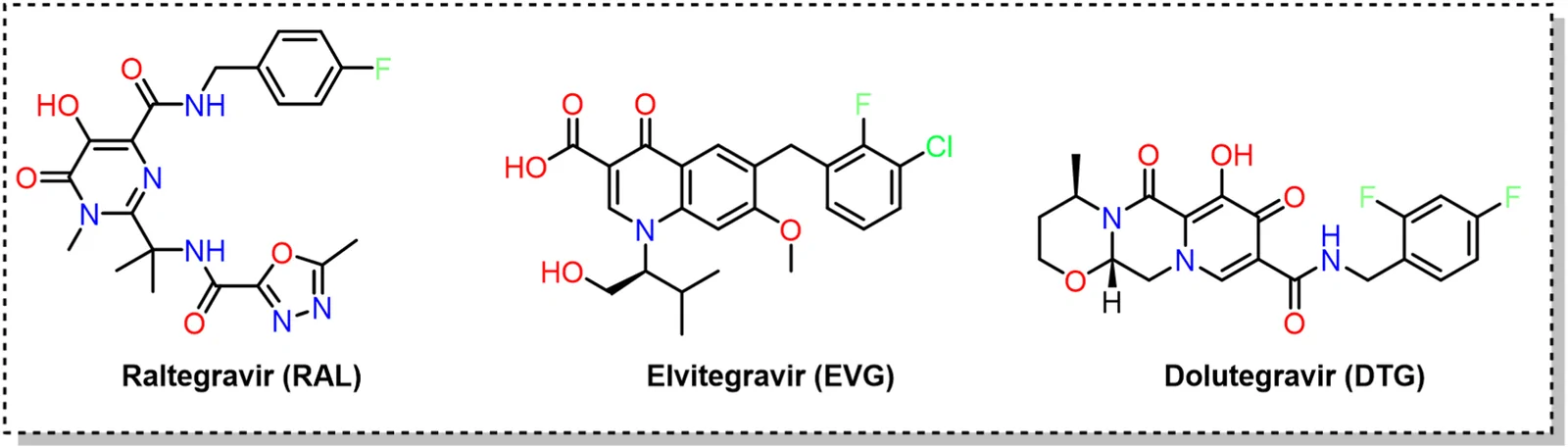
Chemical structures of U.S. FDA-approved HIV-1 IN inhibitors.

In addition to PR, HIV-1 IN plays a crucial role in the viral life cycle by mediating the integration of the double-stranded DNA, which is reverse-transcribed from the viral RNA genome, into the host cell's genome. Since integration is indispensable for viral replication, IN has emerged as a prime therapeutic target in the fight against HIV infections. To date, three HIV-1 IN inhibitors have been approved by FDA: raltegravir (RAL), elvitegravir (EVG), and dolutegravir (DTG). However, resistance mutations to RAL and EVG emerge rapidly, and substantial cross-resistance has been demonstrated between these agents. Although no clinical resistance to DTG or DTG-associated mutations has been reported in treatment-naive patients, limited cross-resistance has been observed between DTG, newer INSTIs, and RAL and EVG (Fig. 7) [41].

**Fig. 8:**
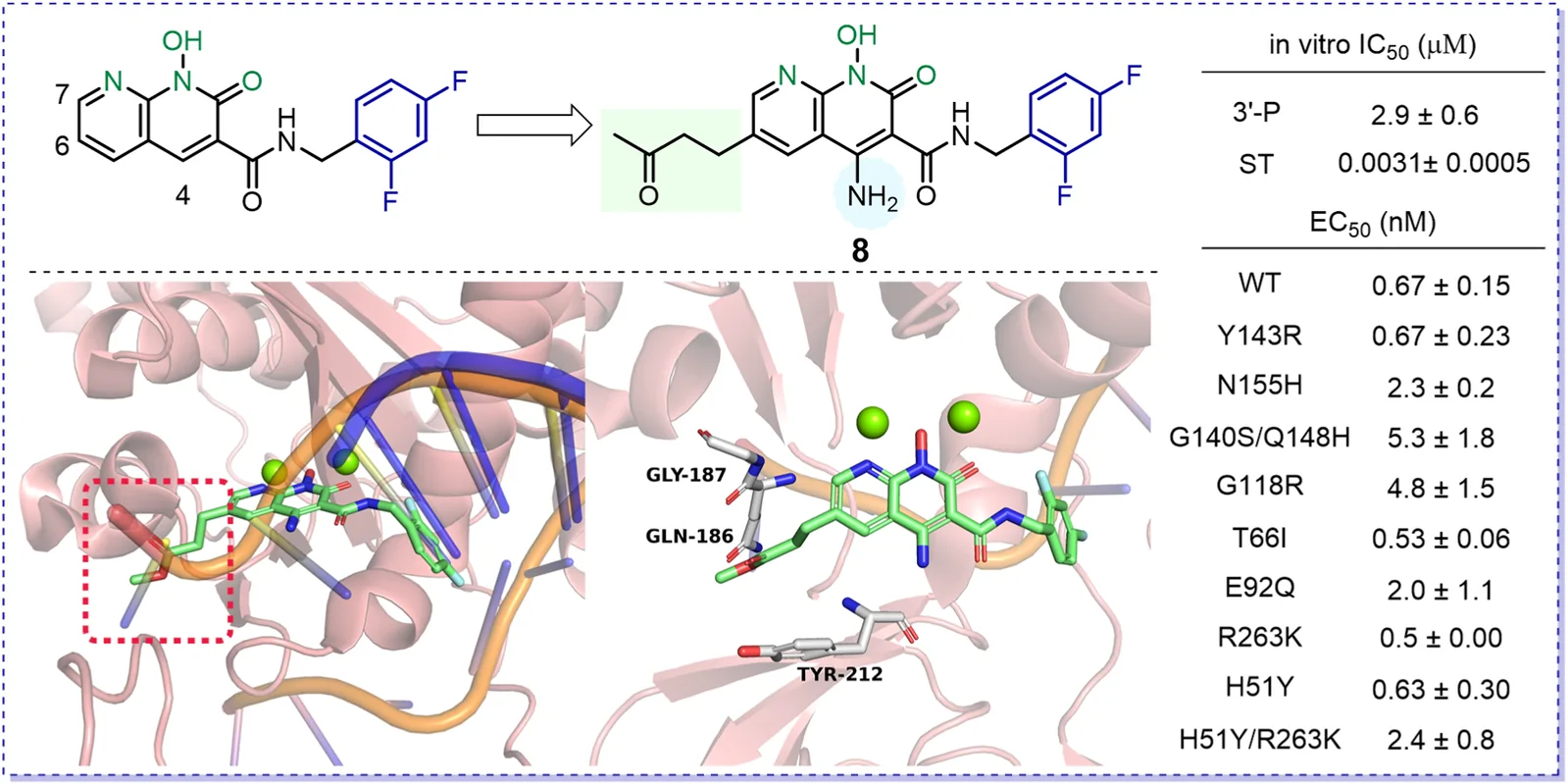
Chemical structure and binding model of compound **8** (PDB code: 5MMB).

Even though the substrate envelope concept originated from studies on PR inhibitors, *Zhao* et al. successfully applied it to antiviral compounds targeting the active sites of integrase (IN). In 2017, they designed a series of novel compounds based on the 1-hydroxy-2-oxo-1,2-dihydro-1,8-naphthyridine-3-carboxamides scaffold. The selection of this scaffold is guided by an analysis of the common structural features shared by the interactions between the three marketed drugs and their respective substrates. By examining the interactions between DTG and its substrates, they found that the tricyclic core of DTG more effectively occupies the substrate cavity and forms a key interaction with G187 of IN. They hypothesized that this interaction might contribute to DTG's enhanced resistance to drug resistance. To achieve more favorable interactions, they presented a comprehensive set of 6-modifications, highlighting the pivotal impact of 6-substituents in preserving antiviral potency against resistant IN mutants. These analogs broadly mimic substrate binding and remain within the substrate envelope upon binding. Compound **8**, featuring a 6-(CH_2_)_2_CO_2_CH_3_ substituent, displayed the most potent antiviral activity across a panel of resistant mutants (Fig. 8). In addition, they obtained the crystal structure of compound **8** bound to the prototype foamy virus (PFV) intasome (Fig. 8). The 6-methyl 3-propanoate side chain of compound **8** resides within a region defined by an envelope formed by both the pre-3′-P viral DNA and the host target DNA substrate. The structure also revealed that the methyl carboxylate group of the compound **8** engages in vdW interactions with the *β*4−*α*2 loop of IN, positioning it between Gln186 and Gly187 residues, as well as the side chain of Tyr212. These findings further corroborate that occupying a space within an envelope defined by the DNA substrates is instrumental in maintaining the broad efficacy of the compounds, informing the foundation for the design and development of next-generation INSTIs that can effectively combat the known drug-resistant mutants.

## 3 Hepatitis C virus (HCV)

HCV is the major etiological agent of chronic liver diseases, affecting approximately 180 million individuals globally. These diseases encompass conditions like cirrhosis, hepatic failure, and hepatocellular carcinoma [42,43]. HCV is a positive-stranded RNA virus within the *Flaviviridae* family and has six genotypes (genotypes 1-6), with types 1 and 3 being the most widespread [44]. Each genotype can further be categorized into multiple subtypes. Current HCV therapies include sofosbuvir, simeprevir, asunaprevir, and daclatasvir, which are used as monotherapy or in combination regimens [[45], [46], [47], [48], [49]]. However, due to the high mutation rate associated with its error-prone RNA replication machinery, drug resistance in HCV develops quickly. Therefore, the development of novel anti-HCV drugs with improved broad-spectrum high resistance barriers is a matter of utmost urgency.

The HCV NS3/4A protease, a serine protease belonging to the chymotrypsin superfamily, features a catalytic triad (His57, Asp81, Ser139) [50]. It consists of an N-terminal serine protease domain and a C-terminal DExH/D-box helicase [51]. As a key therapeutic target, the NS3/4A protease is responsible for cleaving four specific sites on the viral polyprotein. Additionally, it can hydrolyze two human protein targets, such as TRIF and MAVS, that are integral components of the innate immune system, thereby interfering with the host's antiviral immune response [[52], [53], [54]]. The activity of NS3/4A protease can be effectively inhibited by protease inhibitors, which form either reversible covalent or non-covalent bonds with its active site. This competitive inhibition efficiently impedes HCV replication, translation, and post-translational polyprotein processing. Moreover, these inhibitors can restore the type I interferon (INF-I) signaling pathway in infected cells, thereby enhancing the host's innate immune [55]. Recently, the introduction of combination therapies involving boceprevir and telaprevir has significantly enhanced treatment outcomes [56]. However, single-point mutations at Arg155, Ala156, and Asp168 residues have been identified during clinical development and are known to confer resistance to nearly all current inhibitors. NS3/4A protease inhibitors, which are structurally modified substrates specifically designed for the NS3/4A protease, exert their inhibitory effects by competitively binding to the enzyme's active site. Therefore, a comprehensive understanding of the substrate binding domain and substrate recognition sequence of proteases, along with the atomic mechanisms behind how single-point mutations lead to resistance, provides valuable insights for designing inhibitors that maintain effectiveness against a broader range of drug-resistant viral strains.

**Fig. 9:**
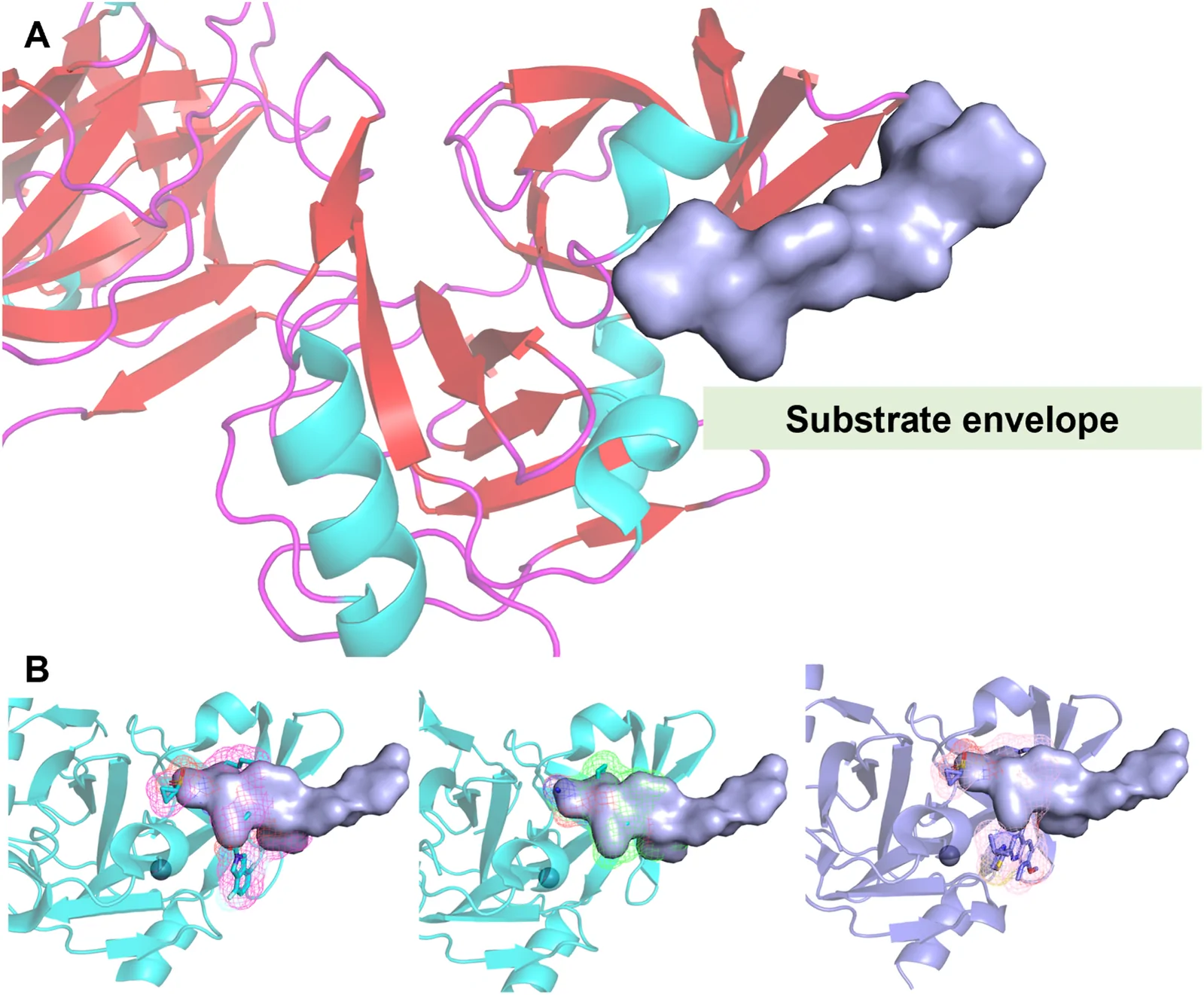
Stereo view of the NS3/4A substrate envelope.

In 2010, Schiffer's group demonstrated the conserved binding of the viral substrate to the protease active site by analyzing the crystal structure of the NS3/4A protease domain, thereby defining the substrate envelope (Fig. 9) [24]. They proposed a universal model to forecast the sensitivity of HCV protease inhibitors to resistance. The model posits that drugs tailored to fit within the substrate envelope are less likely to encounter resistance, as mutations impacting inhibitor binding would also impair the recognition of the viral substrate. This study not only provides a structural basis for understanding the resistance mechanisms of HCV NS3/4A protease inhibitors but also offers novel strategies for designing more effective anti-HCV drugs. By considering the interactions between drugs and the substrate envelope, it is possible to develop new HCV inhibitors that are less prone to resistance.

**Fig. 10:**
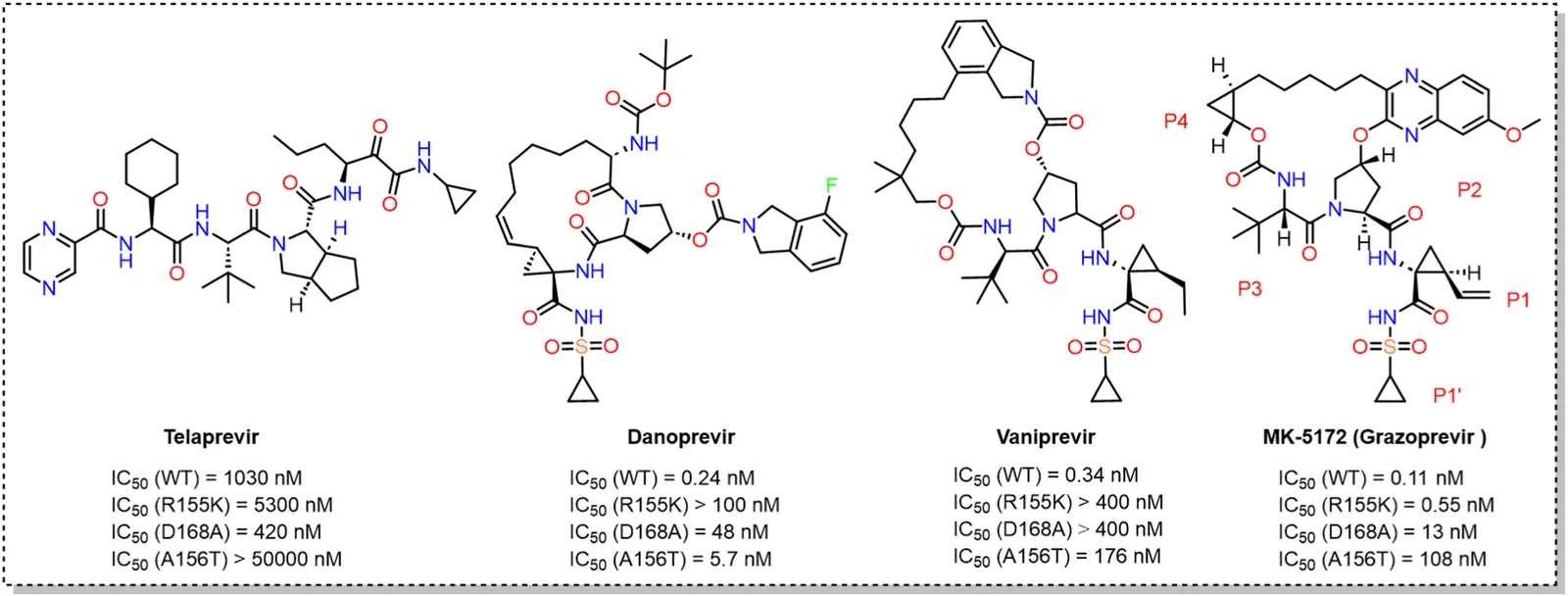
Chemical structures of **Telaprevir**, **Danoprevir**, **Vaniprevir**, and **MK-5172** (**Grazoprevir**). P1–P4 denote the key structural moieties of the inhibitor, which respectively occupy distinct substrate-binding subpockets within the active site of the viral protease. This design ensures a tight fit within the enzyme's substrate envelope to circumvent viral mutational escape.

In 2012, they conducted inhibition experiments based on viral replication to evaluate the drug activities of **telaprevir**, **danoprevir**, **vaniprevir** and **MK-5172** (grazoprevir) against both the wild-type protease and the drug-resistant viral variants R155K, D168A, and A156T (Fig. 10) [23].

**Fig. 11:**
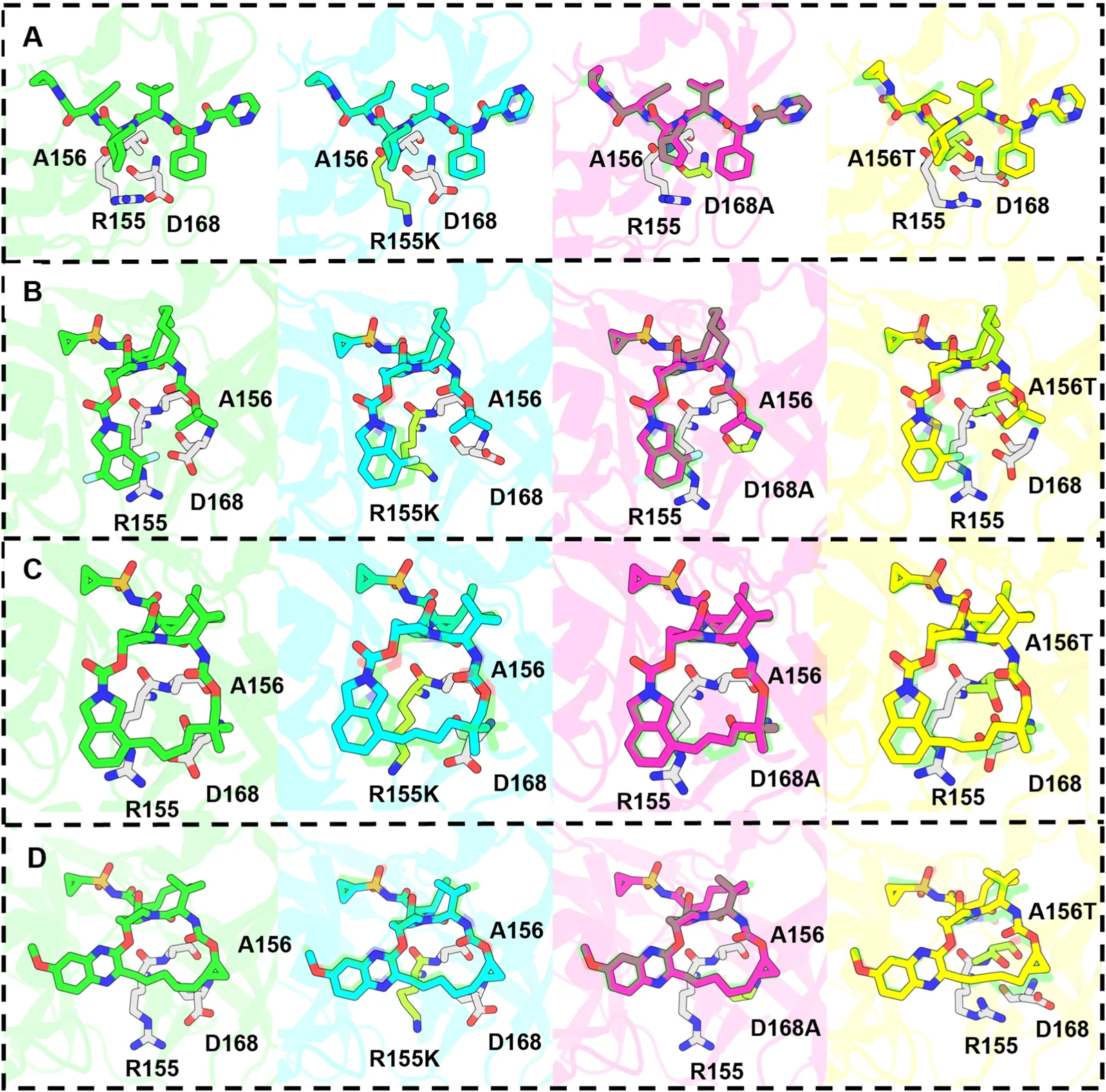
The binding conformations of **Telaprevir**, **Danoprevir**, **Vaniprevir** and **MK-5172** in complex with wild-type protease and several mutant proteases. The protein is shown in cartoon form. Several key amino acid residues are shown in sticks. (A) Stereo view of the **Telaprevir** complexes, (B) **Danoprevir**, (C) **Vaniprevir** and (D) **MK-5172**. The transparent coordinates representing the wild-type structure to better highlight the molecular changes of each mutation.

They further elucidated the molecular mechanism underlying drug resistance against HCV NS3/4A protease inhibitors by analyzing 16 high-resolution crystal structures of these drugs in complex with both the wild-type protease, including three drug-resistant viral variants (Fig. 11). Indeed, drug-resistant mutations predominantly occur in regions where the drugs extend beyond the substrate envelope (Fig. 11A–C), selectively disrupting drug binding with minimal effect on substrate proteolysis. Resistance to protease inhibitors is primarily triggered by mutations that interfere with the electrostatic interaction between Arg155 and Asp168. The P2 moiety of protease inhibitors, located outside the substrate binding region, forms extensive interactions with Arg155, Ala156, and Asp168 residues. Thus, the flexibility of the P2 structure affects drug sensitivity towards different mutants. **MK-5172** adopts a novel binding conformation in which the P2 quinoxaline moiety residues distant to S2 subsite and interacts with the catalytic residues His57 and Asp81, without direct engagement of Arg155 or Asp168 (Fig. 11D). This unique binding pattern explains its potent inhibitory activity against R155K and D168A variants. Crystal structures illustrated that flexible P2 substituents, which lack the P2-P4 macrocycles, such as those in danoprevir, can counteract potency losses due to A156T and D168A mutations. Specifically, the P2 moiety, located outside the substrate envelope, can adapt through intrinsic flexibility and maintain essential interactions with catalytic residues, thereby attenuating the susceptibility of protease inhibitors to Arg155, Asp168, and A156T mutations. These insights offer a pivotal foundation for guiding future strategies in protease inhibitors design.

**Fig. 12:**
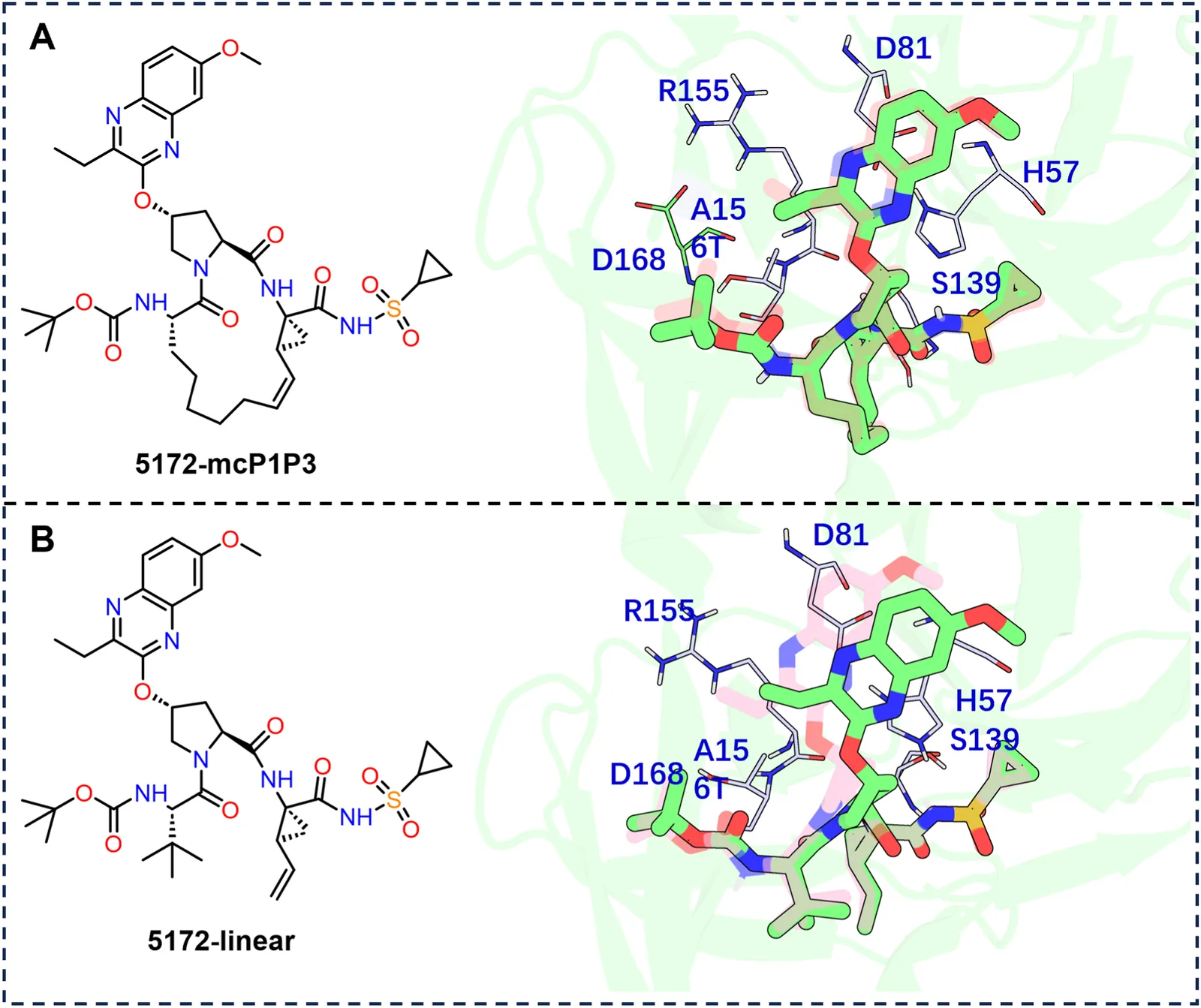
(A) The chemical structures of compound **5172-mcP1P3** and its binding mode in superimposed crystal structures of WT (PDB code: 5EPN) and A156T protease (PDB code: 5EPY). The transparent conformation corresponds to the binding mode in the wild-type enzyme. (B) The chemical structures of compound **5172-linear** and its binding mode in superimposed crystal structures of WT (PDB code: 5EQQ) and A156T protease (PDB code: 5ETX). The transparent conformation corresponds to the binding mode in the wild-type enzyme.

Although **MK-5172 (grazoprevir)** maintained strong inhibitory activity against R155K and D168A mutants, its efficacy is markedly diminished against the A156T mutant. This can be attributed to the strong rigidity imparted by the P2-P4 macrocycle in MK-5172 (grazoprevir), which, the P2 quinoxaline forms critical interactions with the H57 and D81 residues, while enhancing potency through critical interactions between the P2 quinoxaline and H57/D81 residues, leads to spatial constraints upon encountering the A156T mutation To investigate the impact of macrocycle removal on both activity and binding mode, Schiffer's group conducted an extensive crystallographic and thermodynamic analysis on the linear and P1-P3 analogs of **MK-5172** [57]. Their results indicate that macrocyclization does not alter the orientation of the quinoxaline group but influences the response to the A156T mutation. In the A156T-**MK-5172** complex, the A156T substitution induces a conformational shift of **MK-5172** (grazoprevir) away from the active site. Simultaneously, the quinoxaline moiety exhibited a partial loss of critical superposition with His57 and Asp81 residues. Without the P2-P4 macrocycle, the inhibitor can adjust its conformation to fit the A156T mutation in both A156T-5172-linear and A156T-5172-mcP1P3 complexes (Fig. 12). The unique binding mode of **MK-5172** (grazoprevir) is also maintained upon removal of the P2-P4 macrocycle. The **5172-mcP1P3** mimic presents an opportunity for further optimization of protease inhibitors that interact with the stable stacking of catalytic residues while remaining within the substrate envelope.

**Fig. 13:**
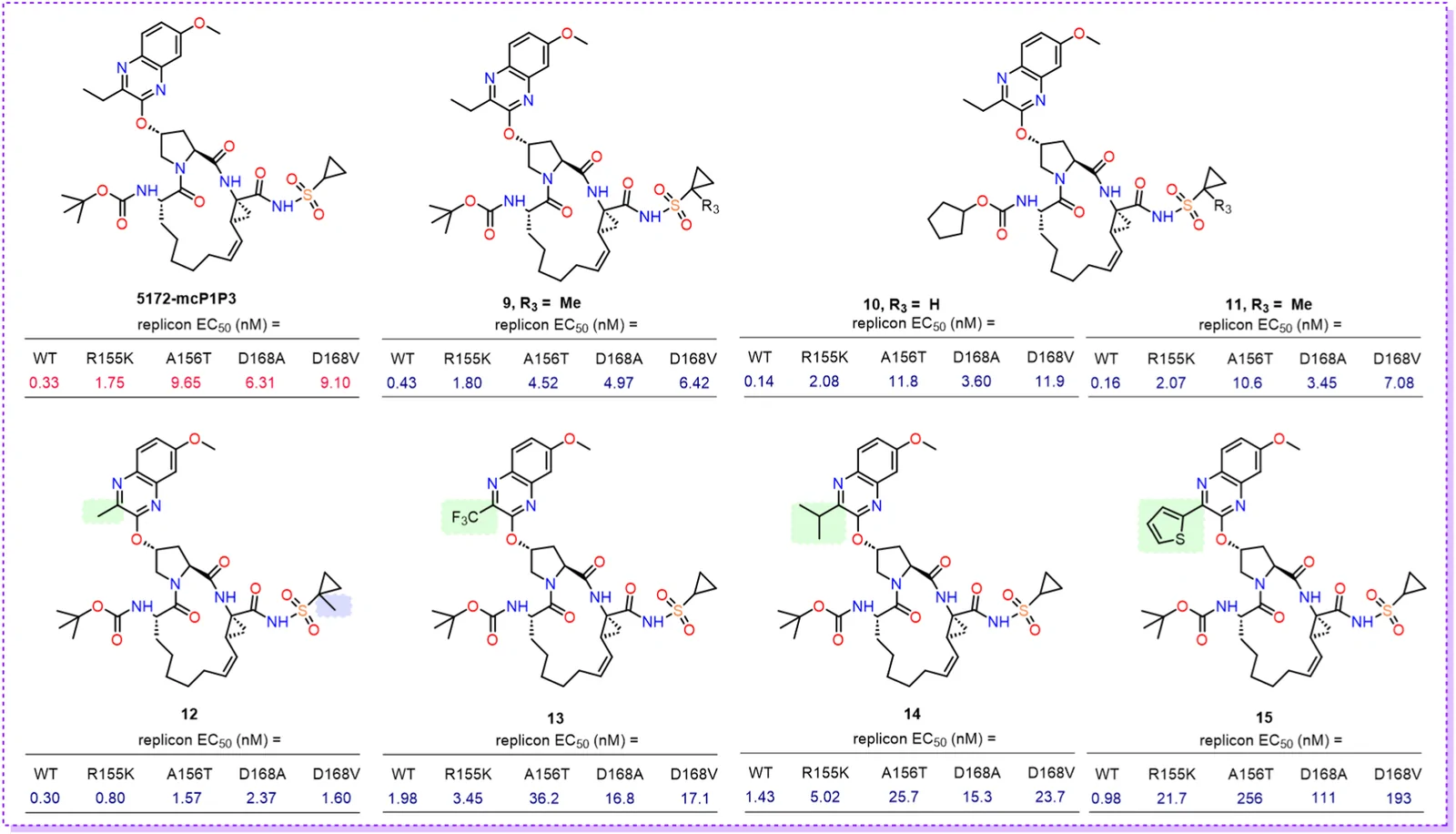
Chemical structures of 5172-mcP1P3 derivatives.

Building on the above research, Schiffer's team designed and synthesized a series of HCV NS3/4A protease inhibitors to further improve the antiviral potency of **5172-mcP1P3** analogs against drug-resistant variants and multiple genotypes [58]. Guided by resistance mechanisms and the substrate envelope principle, these inhibitors incorporated a P2 quinoxaline scaffold bearing diverse substituents at the 3-position and showed potent activity against major resistant HCV strains. Systematic structure-activity relationship (SAR) investigations were performed by modifying the P1′ group and N-terminal P4 capping group (Fig. 13). Replacing the P1′ cyclopropylsulfonamide with 1-methylcyclopropylsulfonamide (compound **9**) maintained or slightly improved potency against wild-type (WT) and resistant viruses. Introduction of a bulkier cyclopentyl P4 capping group (compounds **10** and **11**) enhanced WT and D168A inhibitory activity while preserving efficacy against R155K and D168V variants. A key SAR trend emerged at the P2 quinoxaline 3-position: reducing the size of the hydrophobic substituent (e.g., ethyl → methyl, compound **12**) markedly improved potency against R155K, A156T, D168A, and D168V.

**Fig. 14:**
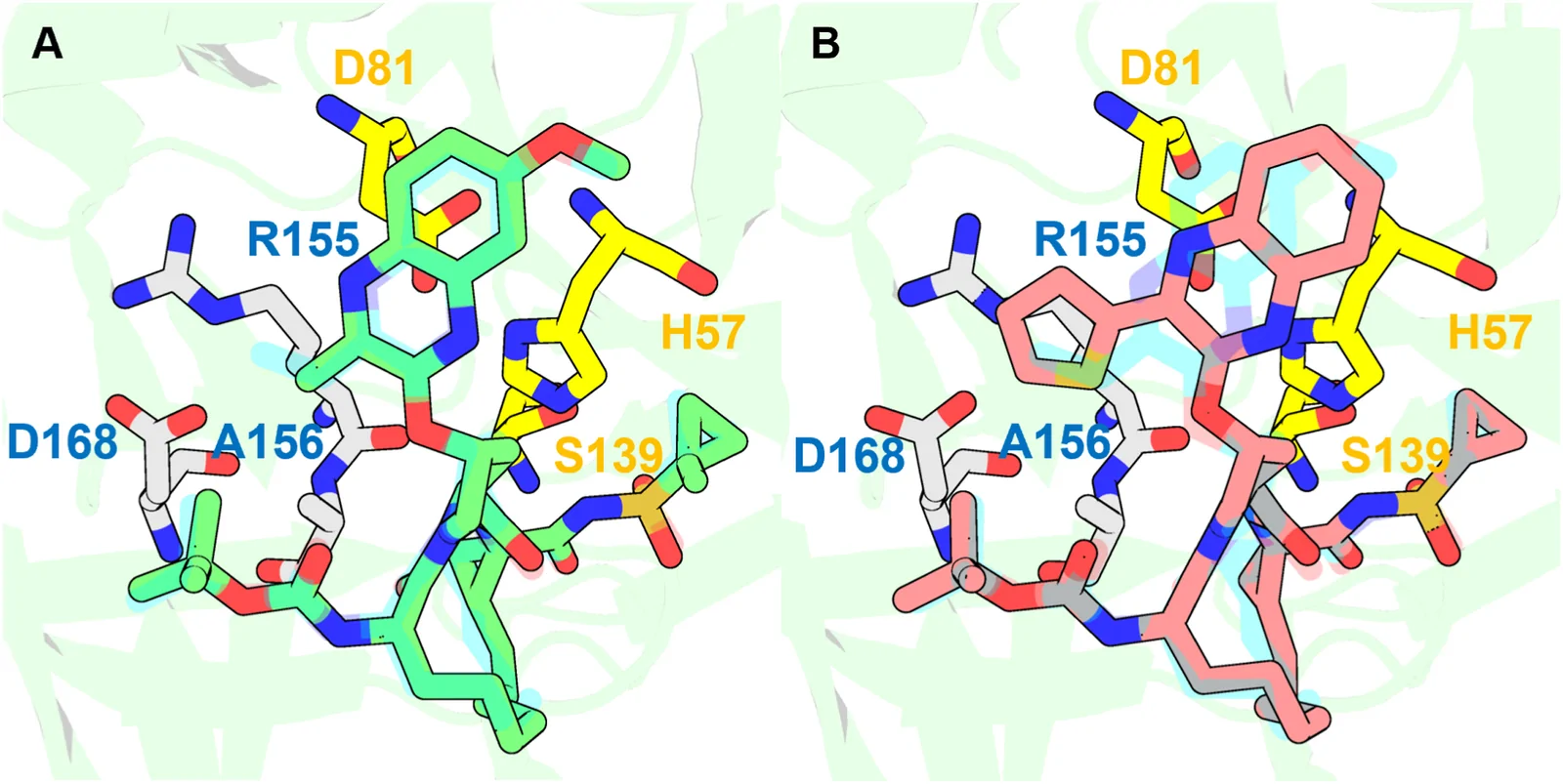
(A) X-ray crystal structure of wild-type1a HCV NS3/4A protease in complex with inhibitor **9** (PDB: 5VOJ) and superposition of wild-type-**5172-mcP1P2** and wild-type-**9** complexes, (B) X-ray crystal structure of wild-type1a HCV NS3/4A protease in complex with inhibitor **15** and superposition of wild-type-5172-mcP1P2 and wild-type-**15** complexes (PDB code: 5VP9). The transparent coordinates represent the **5172-mcP1P2**.

Crystal structures confirmed that the smaller methyl group preserved favorable hydrophobic contacts with Ala156 while reducing steric hindrance with the bulkier Thr side chain in the A156T mutant (Fig. 14). In contrast, installing a larger, electron-withdrawing trifluoromethyl group (compound **13**) or a bulky thiophene group (compounds **14** and **15**) severely impaired activity against A156T and D168A variants. Structural comparisons revealed that large 3-substituents forced the quinoxaline away from the catalytic triad, disrupted key π-stacking interactions, and created excessive contacts with the S2 subsite. The A156T mutation further introduced severe steric clashes with bulky groups such as thiophene. These results demonstrate that inhibitors relying on extensive interactions with S2 subsite residues are highly susceptible to mutations at Arg155, Ala156, and Asp168.

Collectively, this study validates a rational design strategy: combining substrate envelope guidance with appropriate conformational flexibility enables the development of next-generation HCV NS3/4A inhibitors with balanced potency and high resistance barriers.

**Fig. 15:**
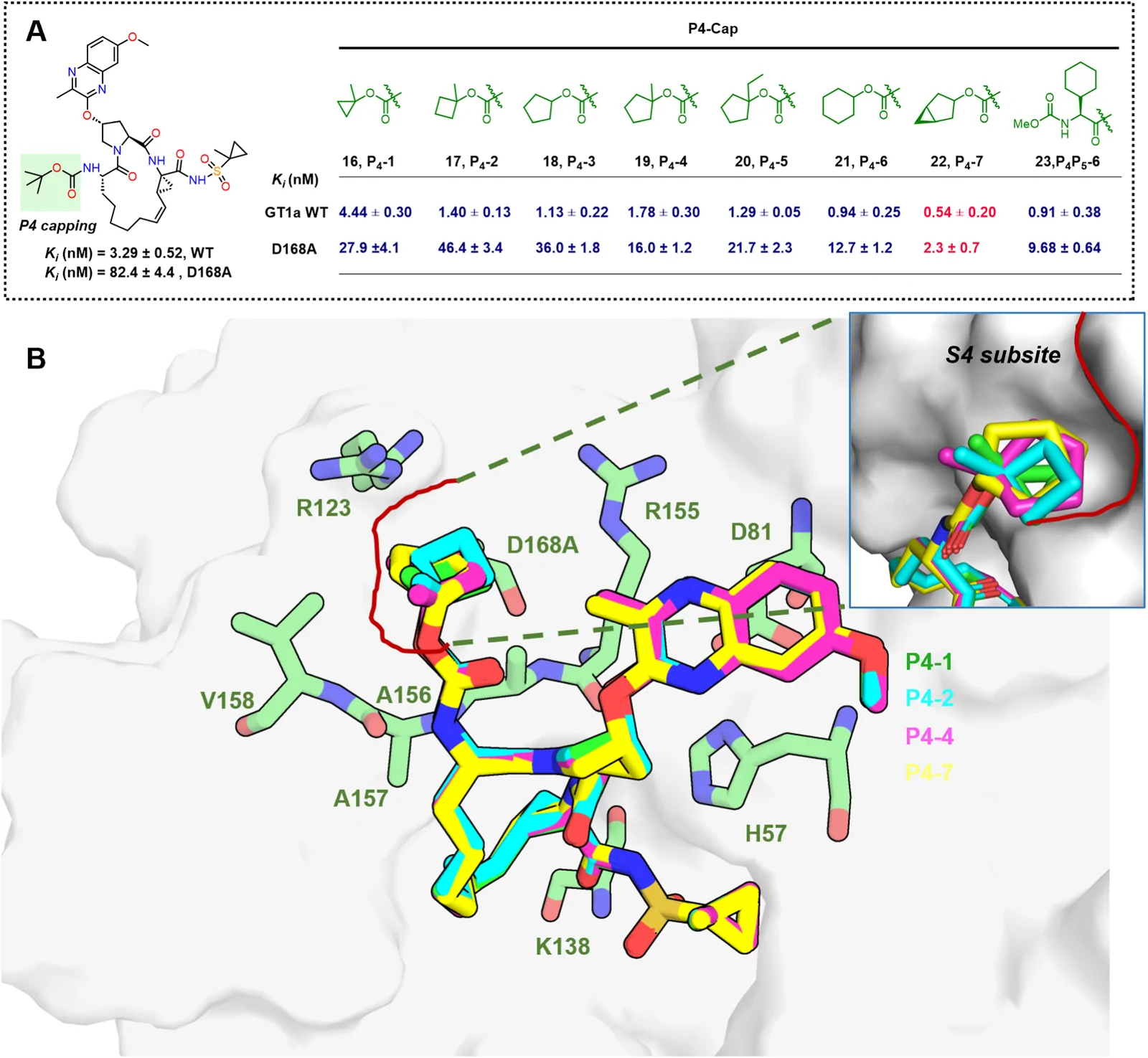
(A) Chemical structures of P4-Cap series compounds. (B) X-ray crystal structures of wild-type1a HCV NS3/4A protease in complex with compounds **16** (P4-1) (PDB: 6PIZ), **17** (P4-2) (PDB: 6PIY), **19** (P4-4) (PDB code: 6PJ1) and **22** (PDB code: 6PIV). The S4 subsite and the binding confirmation of the inhibitor at the S4 subsite were highlighted.

The aforementioned research characterized the role of P2 moiety in determining the potency and resistance profile of the inhibitors. In 2020, Schiffer's group designed and synthesized a series of inhibitors with modifications and extensions in the P4 direction proximal to Asp168. Two sets of modifications were incorporated. One involved capping of the modified P4 (P_4_ series), and the other involved extending the P4 part to mimic substrate interaction up to the P5 position (P_4_P_5_ series), aiming to systematically investigate the impact of size and shape variations in the P4 moiety on potency profiles (Fig. 15A) [59]. The P4-series inhibitors showed a potency range of 4 to 0.5 nM against the wild-type protease in enzyme inhibition assays. Among them, the most potent compound, compound **22** (**P_4_-7)**, exhibited a comparable potency to that of grazoprevir against wild-type proteases (0.54 ± 0.20 nM), and demonstrated a 20-fold higher potency than grazoprevir against D168A variants (2.3 ± 0.70 nM). The P_4_P_5_ series inhibitors were less potent than the P4 series, with potencies ranging from 1 to 30 nM against the wild-type and 10 to 900 nM against the D168A protease. Compound **23** was the most potent in this series, with potencies of 0.91 ± 0.38 nM against the wild type and 9.68 ± 0.64 nM against the D168A variant.

Based on the determined co-crystal structure, they further elucidated the molecular basis underlying enhanced efficacy and identified reasons for reduced potency in certain designed inhibitors. Both series successfully exploited the untapped potential within the substrate envelope, conferring a competitive edge over grazoprevir and the progenitor compound. The binding conformation of inhibitors **16**, **17**, **19**, **22** exhibited pronounced congruence (Fig. 15B). The study revealed that the optimal occupation of the S4 pocket is essential for the potency of inhibitors. Inhibitors manifesting suboptimal efficacy commonly exhibit insufficient engagement of the S4 pocket, culminating in incomplete and non-optimal binding interactions. Generally, the rational design of inhibitors can leverage the evolutionarily conserved regions of the target, thereby enhancing both their inhibitory effectiveness and their resistance profiles against drug resistance.

**Fig. 16:**
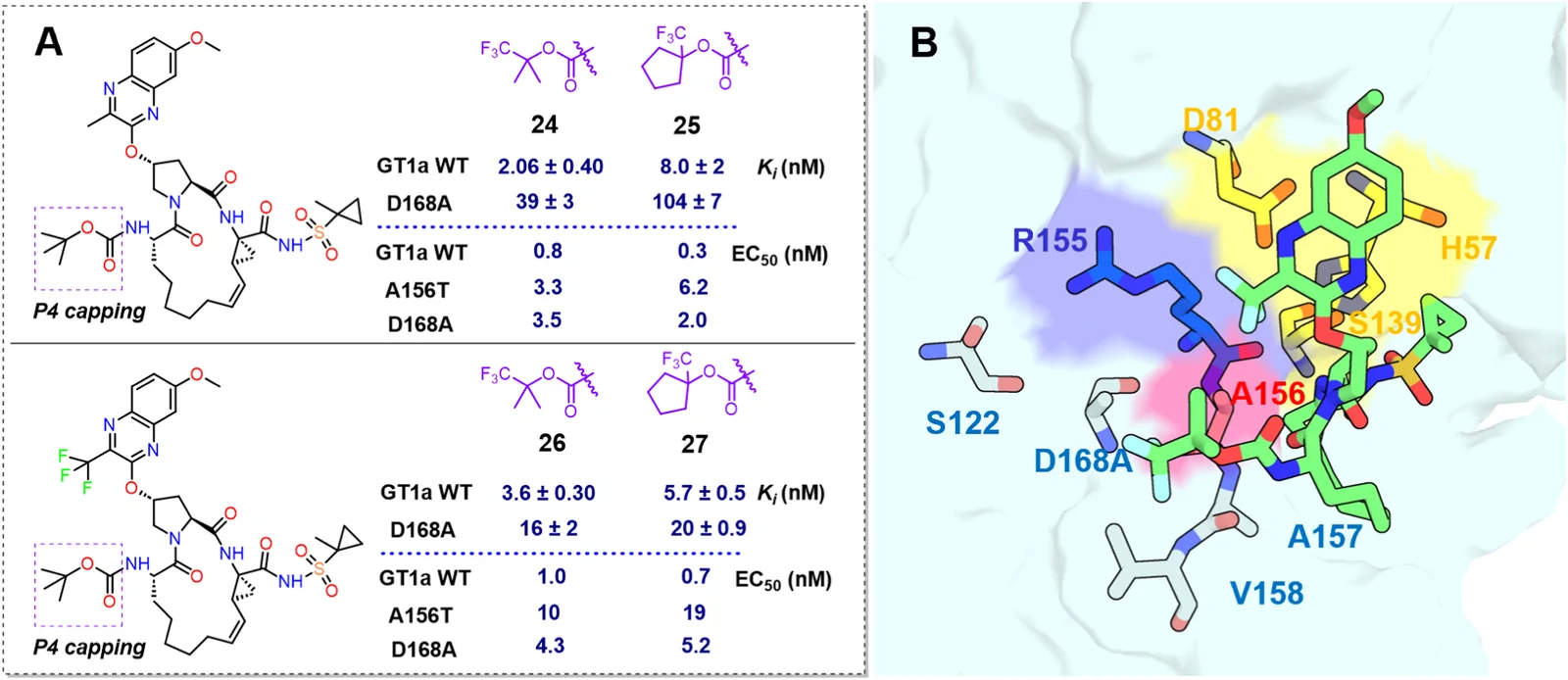
(A) Chemical structures of compounds **24- 27**. (B) Crystal structures of compound **26** in complex with the D168A NS3/4A protease variant (PDB: 7L7L).

Utilizing structure-guided design strategy, the research team delved deeper into the impact of the interaction between the P4 capping group and a portion of the P2 heterocyclic moiety on the potency profiles against resistant variants [60]. They conducted investigations on two series of inhibitors, incorporating distinct P2 quinoxaline moieties and diverse P4 capping groups, encompassing both acyclic and cyclic functionalities, with or without fluorine substitution, resulting in compounds **24-27**. It was discovered that the 3-methylquinolones exhibited outstanding potency profiles with EC_50_ values below 5 nM against D168A variant. Almost all of the compounds maintained low nanomolar antiviral activities against A156T variant, which were significantly more potent than those of two clinically approved drugs, grazoprevir and glecaprevir. The SARs analysis demonstrated the exceptional capability of fluorinated P4 groups to sustain favorable interactions within the S4 pocket. The co-crystal structure of compound **26** complexed with the D168A protease variant revealed that one of the P4 geminal methyl groups is oriented towards the trifluoromethyl group of the P2 quinoxaline, and the other is directed away from the protein surface (Fig. 16). The trifluoromethyl group occupies the S4 pocket, making contacts with Arg123, Val158, and Asp168 residues. These strengthened interactions within the S4 pocket are presumably the underlying cause for the remarkable antiviral activity of compound **26** against drug-resistant HCV variants. Generally speaking, fine-tuning the P4 modification can boost the efficacy of inhibitors against drug-resistant HCV variant, thereby yielding compounds with potential broad-spectrum potency profiles.

Despite the development of numerous clinical anti-HCV drugs, challenges remain. Notably, there is a lack of pan-genotypic drugs with high resistance potency. Concerning the NS3/4A protease, mutations often occur at residues that extend widely from the substrate envelope. These variants can selectively bind to substrates and evade inhibitor binding. Consequently, designing drugs targeting the substrate envelope can alleviate the emergence of drug resistance. Based on research results, modifications of the P4 moiety and P2 quinoxaline moiety within the P1-P3 macrocycle structure have shown excellent potency against variants. The most prevalent modification entails adding a methoxy group at the 3-position in the P2 quinoxaline moiety. For the P4 moiety, small hydrophobic groups such as cyclopentyl or *tert-*butyl groups are favorable choices. Additionally, fluorine substituents in the P4 capping group also exhibit significant effects. In general, following the substrate envelope-guided design strategy, the key to maintaining high anti-HCV activity and avoiding susceptibility to resistant mutations lies in exerting optimal conformational constraint to enhance potency while allowing for adaptability. This approach endeavors to strike a balance between specificity and flexibility, ensuring that the drug remains effective against a broad range of viral strains.

## 4 Influenza virus

IFV has long been recognized as one of the most prevalent epidemic diseases, exerting a profound impact on human life and health. Even with the availability of seasonal vaccines, controlling IFV remains a formidable challenge [61]. However, for individuals with compromised immune function, these vaccines exhibit limited efficacy; hence, it is imperative to explore drugs targeting specific receptors.

Neuraminidase inhibitors (NAIs) represent the primary therapeutic approach for infections. To date, four types of NAIs, namely Oseltamivir, Zanamivir, Peramivir and Laninamivir, have been approved for therapeutic use and served as the major therapeutic options in clinical treatment. Nevertheless, the extensive use of these drugs has led to the emergence of drug resistance, which significantly restricts their therapeutic efficacy. Therefore, a thorough investigation into the molecular mechanisms underlying drug resistance is crucial for overcoming this obstacle.

NA enables the release of the viral particles from the host cell via catalyzing cleavage of α-glycosidic linkage of terminal sialic acid residues in glycoconjugates [62]. Phylogenetic analysis classifies the IFV A neuraminidases (N1-N9 subtypes) into two groups: being NAs (N1 NA) and NAs (N2 NA) [63]. Despite the active site residues sharing 94% identity between N1 and N2 NAs, the resistance mutations clinically observed for N1 and N2 NAs differ. In 2016, Celia A's group performed structural and dynamic analysis on complexes of N1 and N2 NA with substrates and NA inhibitors. They applied substrate envelope-guided strategy to explain the reasons behind the selection of distinct resistance mutations in IFV N1 NA and N2 NA [64].

The residue Glu119 exhibits a higher mutability in N2 compared to N1, as its interaction with the substrate is analogous to that with inhibitors in N1. As a result of the extensive exposure of Glu119 to N1 substrates, mutations at this locus incur a significant fitness penalty. Conversely, in N2 NA, the interaction of Glu119 with inhibitors is characterized by stronger vdW contacts and hydrogen-bonding interactions than its interaction with substrates, elucidating the propensity of Glu119 mutation without compromising substrate recognition. The aforesaid findings also serve to elucidate the predominant drug-resistant mutations in N1 NA, namely Ser246 and Ile222, as opposed to those in N2. Regarding H274Y, a primary oseltamivir resistance mutation in N1 NA, from the perspective of vdW contact, it was confirmed that the alteration in intermolecular contact at the active site of Glu276 was associated with the susceptibility to H274Y mutation. Oseltamivir, when compared to the substrates and zanamivir, exhibited a significantly stronger average vdW contact with residue Glu276. Consequently, the H274Y mutation that further pushes the Glu276 side chain into the active site will have varying degrees of impact on oseltamivir binding and lead to drug resistance. In summary, the disparities in molecular interactions between NA inhibitors and substrates, especially vdW contacts, can effectively account for the selection of drug-resistant mutations in these two subtypes. Gaining a deeper insight into these interactions offers a fresh perspective for the design of novel NA inhibitors. To minimize the occurrence of drug resistance, the design of novel inhibitors should steer clear of excessive interactions with residues that are liable to mutate. Instead, it should enhance interactions with residues that have more extensive contacts with substrates. Additionally, the inhibitors should more accurately mimic the dynamics and intermolecular interactions of substrates at the active site. Moreover, the flexibility of substrates may endow them with the ability to more easily adapt to the altered active site resulting from mutations. By more closely mimicking substrate kinetics and intermolecular interactions and avoiding contacts that increase susceptibility to resistance, it is conducive to the development of inhibitors with a higher resistance threshold.

**Fig. 17:**
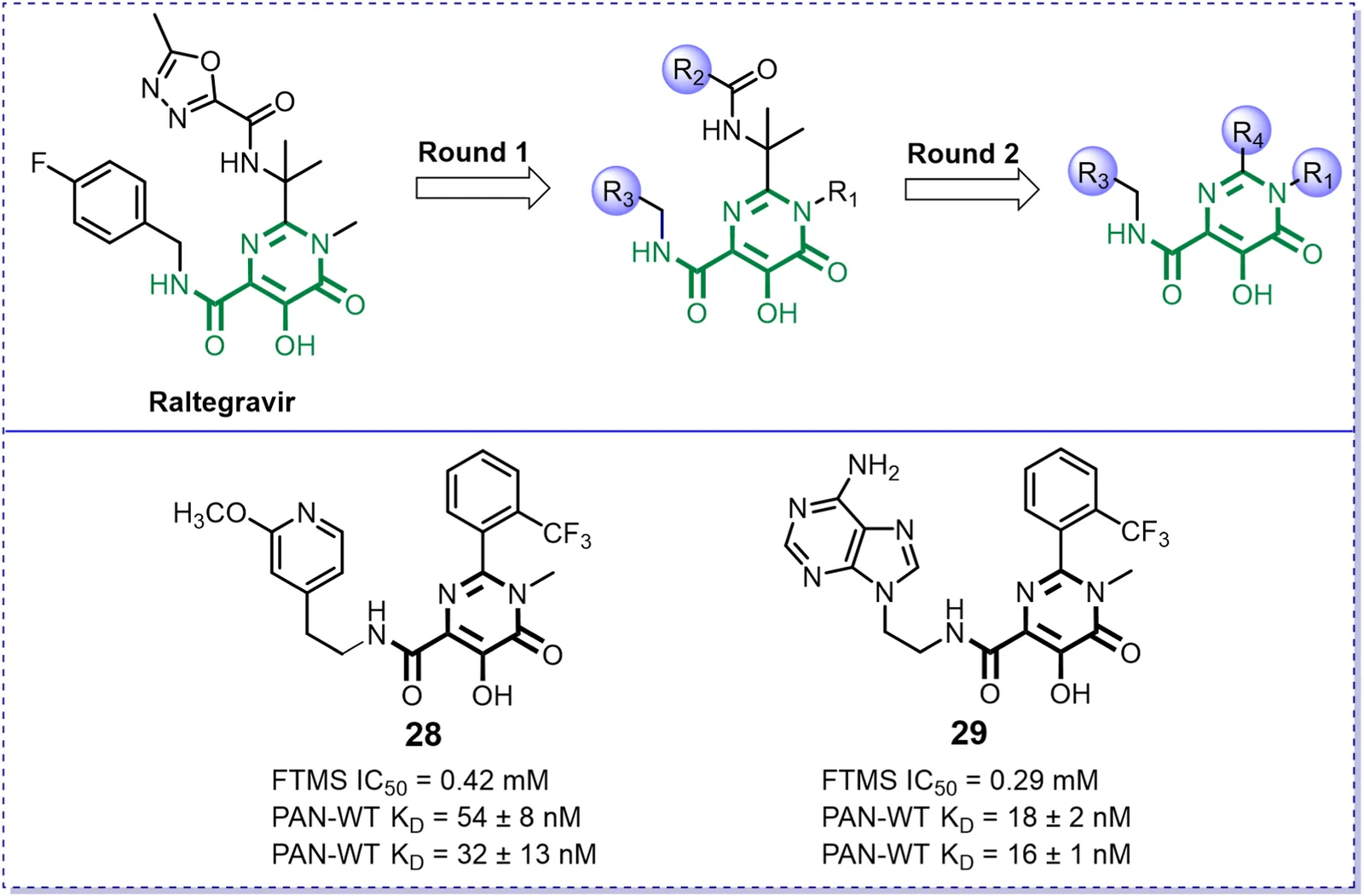
Chemical structures of compounds **28** and **29**.

The successful approval of baloxavir highlights the endonuclease domain (PA_N_) of the RNA-dependent RNA polymerase (RdRp) of IFV as an excellent target for antiviral therapy. Despite baloxavir having demonstrated remarkable efficacy as a first-in-class therapeutic, the rapid emergence of mutants with reduced susceptibility in both preclinical and clinical settings is a concern [65]. To develop novel PA inhibitors with enhanced resistance profiles, Peter J. et al. Employed structure-guided approaches and a substrate envelope-guided design strategy to rationally design derivative molecules [66]. They generated a series of compounds that structurally mimic the bound RNA substrates, with the aim of mitigating the emergence of drug resistance by selectively targeting conserved residues essential for substrate recognition. Using raltegravir as the initial lead compound, they carried out multiple rounds of chemical structure modifications. This was based on the resolved co-crystal structure of raltegravir with PA_N_, resulting in the acquisition of compounds **28** and **29** (Fig. 17). These compounds exhibited submicromolar potency in inhibiting PA_N_. Subsequently, a microscale thermophoresis (MST) assay was conducted to evaluate the *K*_*D*_ values of these compounds with both the wild-type protein and the I38T mutant. As anticipated, the binding of compounds **28** and **29** to I38T remained unaffected by the mutation (PA_N_ -wild-type: *K*_*D*_ = 54 ± 8 nM, 18 ± 2 nM, respectively; PA_N_-I38T: *K*_*D*_ = 32 ± 13 and 16 ± 1 nM, respectively).

**Fig. 18:**
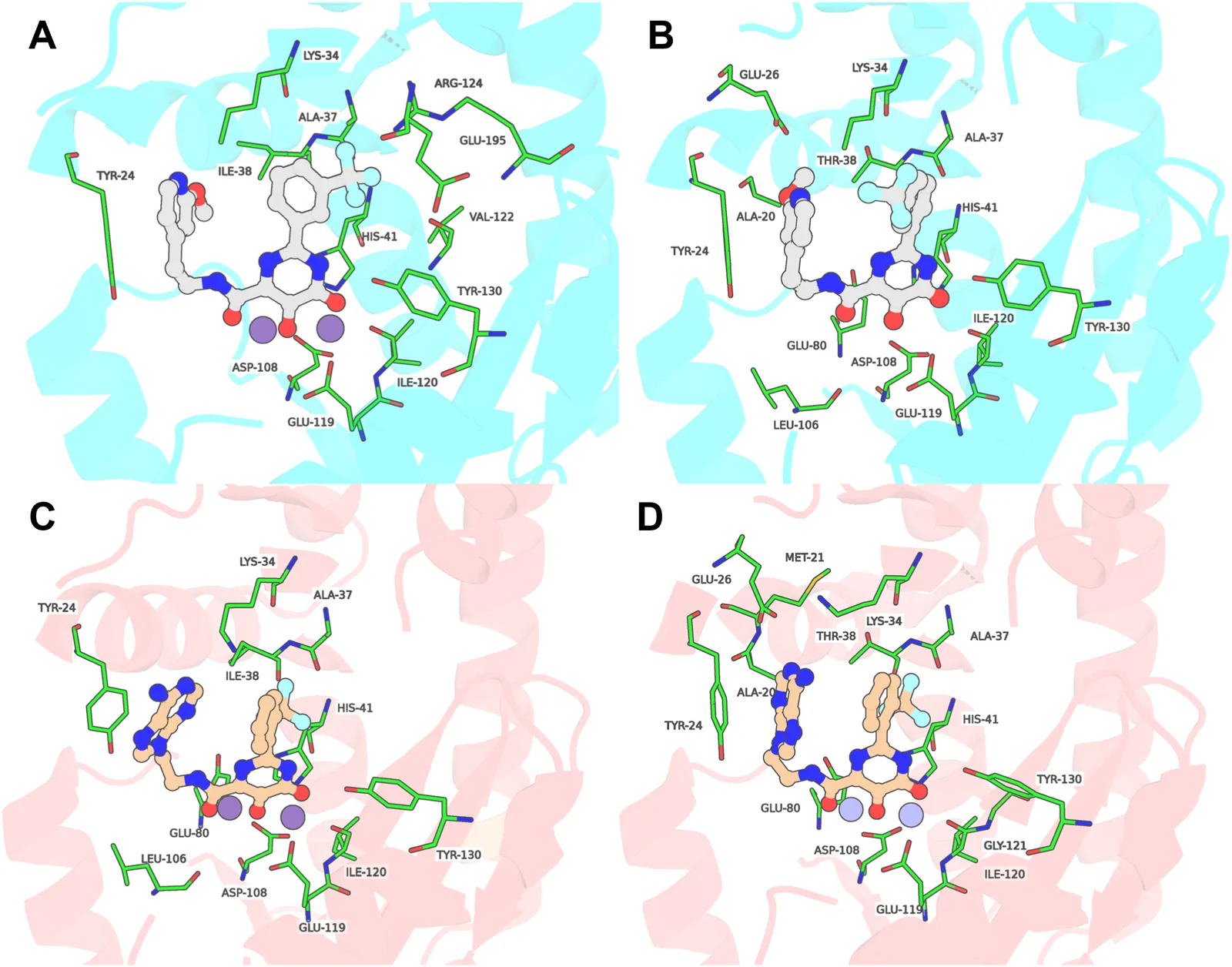
(A, B) X-ray crystal structures of wild-type PA (PDB code: 8DPJ) and I38T mutant (PDB code: 8DIP) in complex with **28** (PDB code: 8DPJ), (C, D) X-ray crystal structures of wild-type PA (PDB code: 6V9E) and I38T (PDB code: 6V6X) mutant in complex with compound **29**.

Furthermore, the crystal structures of the wild-type PAN and the I38T mutant in complex with these compounds were determined. The structural analysis revealed that the central pyrimidinone-carboxamide moiety indeed coordinates the two metal ions at the active site. The left-side moiety forms effective π-π stacking and hydrogen-bonding interactions with Tyr24 and Glu26, respectively. The 2-CF_3_-phenyl occupied a cavity afforded by Ala37 and was bordered by Ile38 and His41 residues. The binding mode of compounds **28** and **29** to the I38T mutant was similar to that in the wild-type PA (Fig. 18A–D). In the complex with compound **28**, the 3-methoxy and 4-pyridyl groups were rotated outwards by 180°. In the complex involving compound **29**, the orientation of the adenosine is significantly different. The structural analysis of the compounds **28** and **29** in their complexes validated their structure-based approach on the basis of substrate envelope, demonstrating effectiveness against the baloxavir-resistant mutant I38T.

## 5 SARS-CoV-2

Coronaviruses (CoVs) constitute a group of non-segmented positive-sense single-stranded RNA viruses. SARS-CoV-2 was first identified as the etiological agent of COVID-19 in December 2019 [67]. The rapid global dissemination of this virus has highlighted the formidable challenges associated with controlling viral transmission and developing effective treatments. This is especially true considering the virus's inherent tendency towards mutation and evolution. The main protease (M^pro^) assumes a pivotal role in the replication process of CoVs by cleaving polyproteins into functional proteins necessary for viral maturation. Additionally, M^pro^ is also involved in interactions with host cells during infection [4,68]. Targeting M^pro^ or polymerases with therapeutic inhibitors has shown promise in combating SARS-CoV-2 variants.

In 2022, Schiffer's group explored the significance of comprehending the substrate envelope of SARS-CoV-2 M^pro^ for predicting and circumventing drug resistance [69]. They determined nine high-resolution co-crystal structures of M^pro^ in complex with substrate peptides and cleavage products, delineating the substrate envelope and pinpointing evolutionarily vulnerable sites prone to resistance mutations. Furthermore, a comprehensive analysis was conducted on the binding profiles of four M^pro^ inhibitors, including the compound currently in clinical use, within the substrate envelope. Their investigation revealed that several promising inhibitors exhibit suboptimal binding profiles by interacting with residues beyond the substrate envelope, particularly Met49, Asn142, Met165, Glu166 and Gln189 residues. This observation implies that SARS-CoV-2 may potentially develop resistance to these agents through mutations at these specific sites.

Indeed, by identifying vulnerable sites within the SARS-CoV-2 M^pro^ that could potentially give rise to drug resistance, the research offers profound insights into the development of potent inhibitors. These inhibitors are expected to maintain a more durable efficacy against the constantly evolving viral pathogen. Overall, this study contributes to the ongoing efforts to develop antiviral therapeutics for SARS-CoV-2 and provides valuable information for predicting and avoiding drug resistance.

## 6 Limitations and applicability

Although the substrate envelope hypothesis provides a robust theoretical framework for designing antiviral drugs against resistance mutations, its universal applicability is limited by the structural and mechanistic diversity of antiviral targets. Critical evaluation indicates that, in certain contexts, strict adherence to the substrate envelope model may not be essential. In systems characterized by significant backbone dynamics or induced-fit mechanisms, rigid adherence to a static envelope may result in suboptimal inhibitor affinity due to a lack of conformational adaptability. Furthermore, this strategy is inherently restricted to orthosteric sites, precluding its application in the design of allosteric inhibitors that function via distal binding. For instance, the evolutionary trajectories of resistance in HCV NS5B polymerase are completely distinct from its substrate processing mechanisms; in such scenarios, the substrate envelope strategy loses its relevance. Similarly, interventions targeting protein-protein interactions (PPIs) fall outside the scope of this framework. These targets typically feature flat interfaces, lack well-defined substrate clefts, and are devoid of the evolutionary constraints essential for catalysis, rendering them more susceptible to distal mutations. Finally, for emerging targets lacking elucidated catalytic mechanisms or available structures of substrate complexes, constructing a reliable substrate envelope model is inherently impractical.

## **7** **Conclusion & authors**’ **viewpoint**

Drug resistance poses a formidable hurdle in the realm of antiviral drug development, as it accentuates the pressing necessity for strategies that can keep ahead of the rapid evolution of viral strains [70]. This comprehensive review delves into the substrate envelope hypothesis, an innovative paradigm that holds promise in crafting potent antiviral agents capable of sidestepping the specter of resistance.

The substrate envelope hypothesis introduces a conceptual framework proposing that viral substrates occupy a consensus volume or assume a particular shape within the enzyme's active site, protease recognizes substrates via an asymmetric three-dimensional shape rather than a specific amino acid sequence—a spatial arrangement that can be elucidated through the analysis of co-crystal structures with natural substrates. When a drug interacts more strongly with regions outside the substrate envelope volume than the substrate does with the enzyme, it is more likely to induce mutations in the target amino acids, this, in turn, triggers drug resistance and compromises the drug's efficacy.

In contrast, within the substrate envelope volume, the interactions are critical for the enzyme–substrate binding and are less prone to mutations. It restricts the conformation of inhibitor molecules strictly within the conserved space occupied by natural substrates, thereby reducing the likelihood of drug resistance mutations. This is because mutations that disrupt inhibitor binding would simultaneously impair the virus's ability to recognize and cleave its natural substrates, drastically diminishing viral viability.

This molecular-level comprehension of substrate recognition and resistance mechanisms is of utmost importance. It lays the foundation for the rational design of inhibitors that can optimally engage the enzyme's active site. By adopting this approach, the aim is to minimize the propensity for drug resistance while preserving or even enhancing the inhibitory potency of antiviral compounds.

We meticulously examine the application of the substrate envelope hypothesis across a spectrum of viral inhibitors, including those targeting HIV, HCV, IFV, and SARS-CoV-2. In the realm of HIV research, the groundbreaking work of the research group led by Celia has successfully demarcated the substrate envelope by the resolution of co-crystal structures. Through rational modification of the compounds and exploration of structure-activity relationships (SAR), the substrate envelope hypothesis has been comprehensively validated, which, the substrate envelope region is critical for substrate recognition, that serves as a blueprint for the development of novel inhibitors that exhibit a decreased vulnerability to resistance.

To clarify the unique positioning and translational value of substrate envelope-guided design in the development of anti-resistance antiviral agents, we systematically compare this structural design strategy with mainstream rational drug design approaches, including conserved residue-directed optimization, covalent inhibitor construction, allosteric ligand development, and polypharmacophore-based molecular design.

Both substrate envelope strategies and conserved residue-focused design seek to elevate viral genetic barriers by engaging functionally indispensable catalytic residues. Still, single-residue anchoring overlooks holistic pocket-ligand geometric matching. Even ligands tightly bound to invariant residues may protrude beyond the substrate envelope, exposing mutable peripheral pockets that facilitate mutational escape—an inherent flaw eliminated by the envelope's global spatial restraints. Covalent ligands deliver exceptional binding potency via irreversible residue conjugation, compensating for the moderate affinity typical of envelope-restricted small molecules. However, electrophilic warheads frequently trigger off-target cytotoxicity; furthermore, covalent attachment sites may map to non-conserved residues, failing to stall resistance emergence. Their bulky reactive moieties also rarely fit within the confined envelope cavity, narrowing their practical utility. Allosteric modulators bind remote regulatory pockets separate from catalytic clefts and do not compete with native substrates, placing them outside the substrate envelope's theoretical framework. Resistance mutations accumulate within allosteric pockets without impairing substrate processing, yielding low genetic barriers, though this modality suits viral targets lacking defined substrate-binding pockets. Multi-target ligands slow mutational selection through synergistic suppression of distinct viral proteins. Unfortunately, this tactic cannot resolve single-domain resistance when each binding motif is mutation-susceptible, and oversized polypharmacophores generally fail to comply with the envelope's compact spatial limits.

No standalone design modality can simultaneously optimize compound potency, biosafety, and anti-mutagenic profiles. For reversible competitive viral enzyme inhibitors, the substrate envelope delivers a universal geometric filtering rule that complements residue-based screening, covalent functionalization, and multi-target construction. Synergistic combination of multiple design logics—such as installing electrophilic warheads at envelope-embedded conserved residues or embedding envelope spatial restraints into polypharmacophore skeletons—can neutralize the drawbacks of individual approaches and yield lead candidates with balanced affinity and robust resistance resilience.

Analogously, in the case of HCV, the substrate envelope of the NS3/4A protease has been precisely delineated, with drugs that align with this envelope demonstrating a reduced incidence of resistance. In the context of IFV, although the substrate envelope hypothesis was originally conceptualized for peptide-processing proteases, its core principle—employing spatial constraints to maintain interactions with essential residues—is fully applicable to neuraminidase (NA). Although NA processes sialic acid substrates rather than peptides, the evolutionary pressure driving resistance remains consistent. Resistance mutations selectively arise in regions where the inhibitor forms stronger van der Waals interactions and hydrogen bonds with the enzyme compared to the natural substrate. This discrepancy confers a selective advantage to mutations that disrupt drug binding without compromising substrate turnover. Building upon this rationale, the substrate envelope strategy has elucidated the differences in resistance mutational profiles between N1 and N2 neuraminidases, thereby facilitating the development of next-generation NA inhibitors with a higher genetic barrier to resistance. When extending this approach to SARS-CoV-2, the substrate envelope of the M^pro^ protease has been scrutinized to anticipate and circumvent drug resistance. This exploration has yielded valuable insights that are instrumental in the development of robust inhibitors capable of withstanding the challenges posed by evolving viral pathogens.

Nevertheless, the substrate envelope hypothesis continues to encounter specific challenges and drawbacks. In the realm of rational drug design, it is of paramount importance to take into account not merely the conserved regions of wild-type proteins and substrate envelopes but also to foresee any susceptible sites that may undergo mutations, weaken inhibitor binding while maintaining substrate recognition ability. This strategy faces a fundamental trade-off between binding affinity and resistance durability. Stringent adherence to the substrate envelope limits molecular size and the extension of functional groups, resulting in relatively weak binding affinity between inhibitors and the protease. Most compounds designed under this principle exhibit only nanomolar activity, far below the picomolar potency of clinical drugs such as darunavir, making direct clinical translation challenging. The flexibility and conformational adaptability of compounds are indispensable factors that must be considered during the design process. An appropriate degree of flexibility can enhance the accommodation of the compound to the target site, thereby increasing the resistance barrier of the compounds. Furthermore, distal mutations (away from the active site) exert a significant role in affecting protein activity. Contrary to conventional hypotheses, distal mutations can not only compensate for original harmful active site mutations but also directly confer drug resistance. Furthermore, the conserved substrate envelope generated by substrate overlap via the crystal structure remains static, whereas the protein's natural recognition of the substrate is a dynamic process. Furthermore, the strategy imposes severe constraints on scaffold and substituent selection, as it is compatible with only a limited set of classic scaffolds and offers few modifiable sites. To fit within the envelope, bulky substituents must be removed, eliminating potential hydrogen-bonding and hydrophobic interactions and further weakening binding capacity.

In addition, the substrate envelope approach cannot circumvent viral compensatory mutations. The virus can maintain substrate recognition through non-active-site compensatory mutations while reducing inhibitor affinity, thereby bypassing the resistance barrier imposed by the substrate envelope.

Therefore, it is imperative to integrate molecular dynamics for a comprehensive analysis of substrate-protein recognition. Future improvements can integrate molecular dynamics simulations and free-energy calculations to dynamically recapitulate conformational changes in the protease and its inhibitors, correct biases in conformational prediction, and achieve simultaneous optimization of binding affinity and substrate envelope compatibility. Meanwhile, researchers can break the limitation of relying on a single scaffold by combining substrate envelope rules with innovative drug-design concepts—including macrocyclization, heterocyclic engineering, and covalent binding—to expand molecular diversity. With the remarkable progress of AI technology, prediction models have emerged as indispensable tools. By combining machine learning with molecular dynamics and experimental data, we can comprehensively explore both the static and dynamic aspects of the substrate envelope to identify key factors determining potency, thereby significantly enhancing efficiency and accuracy.

In conclusion, the substrate envelope hypothesis emerges as an invaluable asset in structure-based drug design (SBDD), offering a systematic methodology for the conception of inhibitors fortified with an enhanced resistance profile. By amalgamating molecular dynamics simulations, machine learning algorithms, and empirical data, the substrate envelope strategy can be further honed to confront the challenges posed by the swift mutation of disease targets. Notably, the substrate envelope strategy, initially validated for enzymatic targets, now extends to non-enzymatic viral proteins, as exemplified by a recent study of “Pseudo substrate Envelope” on HIV capsid modulation [71]. Looking ahead, the integration of the substrate envelope hypothesis with cutting-edge computational techniques and robust experimental validation will not only bolster the armory against existing viral threats but also fortify the pharmaceutical industry's readiness to address future viral resistance challenges.

## CRediT authorship contribution statement

**Qiaojie Xu:** Writing – original draft, Visualization. **Jiwei Zhang:** Writing – original draft, Visualization. **Yanxu Hou:** Visualization. **Zhou Tan:** Visualization. **Heng Gao:** Visualization. **Edeildo F. Silva-Júnior:** Writing – review & editing. **Shenghua Gao:** Writing – review & editing, Supervision. **Peng Zhan:** Writing – review & editing, Supervision, Funding acquisition, Conceptualization. **Xinyong Liu:** Writing – review & editing, Supervision, Funding acquisition, Conceptualization.

## Ethics approval

Not applicable.

## Data availability

Not applicable.

## Declaration of generative AI in scientific writing

Not applicable.

## Funding information

We gratefully acknowledge financial support from Key Research and Development Program, Ministry of Science and Technology of the People's Republic of China (2023YFC2606500; 2023YFE0206500), and Shandong Laboratory Program (SYS202205), and the Independent Project of the Shandong Basic Research Special Zone (Pharmacy) (TQ052025001). Open Research Projects of the Shandong Provincial Key Laboratory of Intelligent Surveillance, Early Warning and Prevention and Control of Infectious Diseases (CDC25KF3).

## Declaration of competing interest

The authors declare no conflict of interest.
